# Assessment of Parent Income and Education, Neighborhood Disadvantage, and Child Brain Structure

**DOI:** 10.1001/jamanetworkopen.2022.26208

**Published:** 2022-08-18

**Authors:** Divyangana Rakesh, Andrew Zalesky, Sarah Whittle

**Affiliations:** 1 Melbourne Neuropsychiatry Centre, Department of Psychiatry, University of Melbourne, Melbourne Health, Melbourne, Victoria, Australia; 2 Melbourne School of Engineering, University of Melbourne, Melbourne, Victoria, Australia

## Abstract

**Importance:**

Although different aspects of socioeconomic status (SES) may represent distinct risk factors for poor mental health in children, knowledge of their differential and synergistic associations with the brain is limited.

**Objective:**

To examine the independent associations between distinct SES factors and child brain structure.

**Design, Setting, and Participants:**

We used baseline data from participants aged 9 to 10 years in the Adolescent Brain Cognitive Development (ABCD) study. These data were collected from 21 US sites between September 2017 and August 2018. Study participants were recruited from schools to create a participant sample that closely reflects the US population.

**Exposures:**

Neighborhood disadvantage was measured using the area deprivation index. We also used data on total parent or caregiver educational attainment (in years) and household income-to-needs ratio.

**Main Outcomes and Measures:**

T1-weighted magnetic resonance imaging was used to assess measures of cortical thickness, surface area, and subcortical volume.

**Results:**

Data from 8862 ABCD participants aged 9 to 10 years were analyzed. The mean (SD) age was 119.1 (7.5) months; there were 4243 girls (47.9%) and 4619 boys (52.1%). Data on race or ethnicity were available for 8857 of 8862 participants: 173 (2.0%) were Asian, 1099 (12.4%) were Black or African American, 1688 (19.1%) were Hispanic, 4967 (56.1%) were White, and 930 (10.5%) reported multiple races or ethnicities. Using 10-fold, within-sample split-half replication, we found that neighborhood disadvantage was associated with lower cortical thickness in the following brain regions (η^2^ = 0.004-0.009): cuneus (*B* [SE] = −0.099 [0.013]; *P* < .001), lateral occipital (*B* [SE] = −0.088 [0.011]; *P* < .001), lateral orbitofrontal (*B* [SE] = −0.072 [0.012]; *P* < .001), lingual (*B* [SE] = −0.104 [0.012]; *P* < .001), paracentral (*B* [SE] = −0.086 [0.012]; *P* < .001), pericalcarine (*B* [SE] = −0.077 [0.012]; *P* < .001), postcentral (*B* [SE] = −0.069 [0.012]; *P* < .001), precentral (*B* [SE] = −0.059 [0.011]; *P* < .001), rostral middle frontal (*B* [SE] = −0.076 [0.011]; *P* < .001), and superior parietal (*B* [SE] = −0.060 [0.011]; *P* < .001). Exploratory analyses showed that the associations of low educational attainment or neighborhood disadvantage and low cortical thickness were attenuated in the presence of a high income-to-needs ratio (η^2^ = 0.003-0.007).

**Conclusions and Relevance:**

The findings of this cross-sectional study suggest that different SES indicators have distinct associations with children’s brain structure. A high income-to-needs ratio may play a protective role in the context of neighborhood disadvantage and low parent or caregiver educational attainment. This study highlights the importance of considering the joint associations of different SES indicators in future work.

## Key Points

**Question:**

What are the associations of parent or caregiver educational attainment, income-to-needs ratio, and neighborhood disadvantage (and their interactions) with brain structure in children?

**Findings:**

In this cross-sectional study of 8862 children aged 9 to 10 years, different indicators of socioeconomic status (SES) had distinct correlations with children’s brain structure. Thus, high income-to-needs ratios may play a protective role in the context of low neighborhood SES and parent or caregiver educational attainment.

**Meaning:**

The results of this study suggest that different facets of the socioeconomic context are independently and synergistically associated with child brain structure.

## Teaser

This cross-sectional study uses data from Adolescent Brain Cognitive Development Study participants aged 9 to 10 years to assess the associations of parent or caregiver income and education, neighborhood disadvantage, and child brain structure.

## Introduction

Socioeconomic status (SES) encompasses different facets of a child’s social environment and represents access to both material and nonmaterial resources. Low SES is associated with cognitive deficits and poor mental and physical health across the lifespan.^1,2,3,4,5,6,7^ It has been posited that neurodevelopmental alterations early in life may mediate the association between low SES and mental health and cognitive outcomes.^8,9,10^ As such, understanding the associations between SES and neurobiological development can enhance investigations into the causes of poor health and functioning.

Socioeconomic status has consistently been shown to be associated with brain structure during childhood and adolescence.^8,9,10,11,12,13,14,15,16,17,18,19,20^ However, findings have been mixed in terms of brain regions and direction of association.^12^ These differences may be a result of SES being operationalized in various ways, including as parental income, parental educational attainment, parental occupational prestige, or neighborhood quality.^12^ These indices of SES are only moderately correlated^21^ and likely represent different aspects of the environment and distinct risk and protective factors. There is also evidence to support independent associations between different SES indicators and children’s mental health^7^ and cognitive performance (eg, reading and mathematics scores^22,23^). These effects may occur as a result of distinct underlying mechanisms. For instance, neighborhood disadvantage may affect brain development because of its association with stress (eg, via exposure to noise and crime) as well as exposure to pollutants and toxins in the environment. These mechanisms may be distinct for low household SES, which may affect brain development through lower cognitive stimulation (eg, less access to material resources in the home, lower semantic input and complexity, and lower quality of parent-child interactions).^24^

Despite this knowledge, few studies have examined independent associations between different SES indicators and the brain by including them in the same model (particularly neighborhood SES, which is rarely included in models with family SES indicators). Previous studies provide some evidence for differential associations between different SES indicators and brain structure.^25,26^ However, the available studies have only investigated a limited number of brain regions, and our review indicated that structural associations are likely to be widespread.^12^ Thus, limitations of existing work prompt the need for further investigation. Furthermore, theories suggest that family and neighborhood SES may jointly affect child development (eg, via additive or protective effects).^27,28^ Recent work from our group has shown that such joint associations are reflected in brain functional connectivity.^29^ Investigating the independent and joint associations between different SES indicators and brain structure may help advance our understanding of the neurobiological mechanisms through which different aspects of SES together influence child development—a question yet to be explored in neuroscience. Knowledge gained from this study will provide insight into the relative importance of different facets of the socioeconomic context (and their joint associations) in influencing child brain development.

Given the gaps in the literature outlined earlier, the goal of this study was to test whether parent or caregiver educational attainment, parent or caregiver income, and neighborhood SES have independent and joint associations with child brain structure. We made some general hypotheses based on our recent systematic review of the literature. Namely, based on consistent findings from previous literature (for a review, see Rakesh and Whittle^12^), we expected all 3 SES indicators to be associated with larger hippocampal, amygdala, and striatal volume as well as the structure of brain regions involved in cognitive function (eg, frontal and parietal cortices). Furthermore, we expected generally more robust associations with cortical surface area than thickness. Moreover, we made some specific hypotheses based on theorized proximal mechanisms linking SES with brain structure. Namely, we hypothesized that because parent educational attainment is posited to play a role in shaping sources of cognitive stimulation and language input and complexity,^24,30^ it would be independently associated with the structure of temporal regions. In addition, because neighborhood disadvantage can be associated with increased stress in children (eg, due to exposure to violent crime),^31,32,33^ it may be independently associated with the morphology of brain regions involved in the body’s stress response (eg, medial prefrontal cortex).^34,35^ Finally, although we expected interactions between the SES indicators (eg, that high parent or caregiver income and educational attainment would mitigate the effects of living in a poor neighborhood), we did not make specific hypotheses about which brain regions would be implicated.

## Methods

This cross-sectional study was preregistered on the Open Science Framework (osf.io/5hqkj). The study followed the Strengthening the Reporting of Observational Studies in Epidemiology (STROBE) reporting guideline.

### Participants

Participants were from the ongoing Adolescent Brain Cognitive Development (ABCD) study. Baseline assessment data (release 3) were collected between September 2017 and August 2018.^36^ This large multisite longitudinal study has recruited more than 11 500 children (aged 9-10 years) to comprehensively characterize psychological and neurobiological development from late childhood to early adulthood.^37,38^ The 21 ABCD study sites are universities and research institutes that were chosen through a competitive grant application procedure, and the metropolitan areas within their catchment areas were chosen based on their demographics (ie, similar to those of the US as a whole). These sites include approximately 20% of 9- to 10-year-olds in the US. Within these sites, public, public charter, and private schools within a 50-mile radius of the data collecting site were identified, and schools from which to recruit participants were randomly selected. Participants were contacted directly through birth registries for twin recruitment. Participants completed clinical interviews, neuroimaging, neurocognitive tests, and surveys.

Race and ethnicity data were included owing to the confounding nature of these demographics and socioeconomic disadvantage among the study cohort. This data was used in sensitivity analyses (described hereinafter). Parents or caregivers were asked 2 questions: (1) “What race do you consider the child to be?” (Asian, Black or African American, White) and (2) “Do you consider the child Hispanic/Latino/Latina?” (yes or no). Based on the responses, a 5-level variable classified as Asian, Hispanic, non-Hispanic Black, non-Hispanic White, or multiracial/multiethnic is included in the ABCD Study Data Releases.

Written informed consent was obtained from all parents or caregivers, and all children provided assent. The rights of participants were protected under the local institutional review boards. After exclusion based on imaging quality control criteria and missing SES data, a total of 8862 children were included in the final sample in the main analysis (ie, 3 SES variables in the same model, as described hereinafter; an inclusion and exclusion flow chart is provided in eMethods 4 in the Supplement). Excluded participants were slightly younger and generally had lower SES.

### SES Measures

#### Neighborhood SES

We used a composite measure of neighborhood disadvantage at the census tract level, the area deprivation index (ADI), based on the participant’s primary residential address. The ADI is based on census data on 17 different factors including income, education, employment, and housing quality and provides rankings of neighborhoods as a national percentile.^39,40^ Higher ADI reflects greater disadvantage.

#### Household SES

Mean parent or caregiver educational attainment (in years) was calculated based on the education level of both parents or caregivers. We used the data for 1 parent or caregiver when data for both were unavailable. The income-to-needs ratio was calculated as the median value of the income band (the bands used by ABCD are provided in eMethods 3 in the Supplement) divided by the federal poverty line for the respective household size. Accordingly, a value of 1 would signify being at the poverty threshold, and values greater than or less than 1 would signify being above and below the threshold, respectively. Distributions and correlations are provided in eMethods 1 and 2 in the Supplement.

### Imaging Acquisition, Preprocessing, and Quality Control

Neuroimaging was conducted with harmonized protocols across sites, using either a 3T Siemens, Phillips, or General Electric magnetic resonance imaging (MRI) scanner with a 32-channel head coil. A 3-dimensional T1-weighted image with 1-mm voxel resolution was acquired for all participants. Preprocessing was performed by the ABCD Data Analysis and Informatics Core using a standardized pipeline (for details and quality control procedures, see Hagler et al^41^). Motion detection and correction software programs were used in real time at the Siemens and GE sites.^42,43^ Both manual and automatic techniques were used to check the data for quality. All images were examined by trained professionals for artifacts and abnormalities. The degree of artifact in the cortical reconstruction of postprocessed pictures was double-rated on a scale from 0 to 3, with 3 signifying the most. Based on the ratings, the technicians made usability recommendations (only images with a rating of 0 were included in this study). The signal-to-noise-ratio and head motion measurements were calculated using automated processes. Based on recommendations by Hagler et al,^41^ we also excluded participants who (1) did not meet the FreeSurfer quality control requirements, (2) had missing quality control data, and (3) were suggested for clinical referrals based on incidental MRI findings from structural MRI analyses. These exclusions resulted in a final sample of 10 454 participants with usable imaging data. For further details on MRI procedures, preprocessing, and specific quality control processes, see Casey et al^37^ and Hagler et al.^41^

FreeSurfer (version 5.3.0) was used for cortical surface reconstruction and parcellation (using the Desikan-Killiany atlas) and subcortical brain segmentation (using the aseg atlas). In our study, we examined cortical thickness and surface area (34 variables each) and subcortical volume (7 variables), resulting in a total of 75 variables. Because there were no predictions of lateralized effects, values for the right and left hemispheres were averaged for analyses.

### Statistical Analysis

To examine the independent effects of the 3 SES indicators on brain structure in our main analysis, we conducted linear mixed-effects models (using the *lme4* package in R version 4.1.3 [R Project for Statistical Computing]) with ADI, parent or caregiver educational attainment, and income-to-needs ratio as predictors (in the same model) and brain structure as the dependent variable (75 variables; in separate models). In our exploratory analyses, we examined 3 subsequent sets of 2-way interactions: ADI × education, ADI × income-to-needs ratio, and education × income-to-needs ratio (lower-order main effects were automatically included). After exclusions based on quality control criteria and missing SES data were complete, the sample sizes were 8862 for the main analysis (3 SES variables in the same model) and 9813, 8866, and 9403 for the exploratory analyses (3 interaction models described earlier), respectively.

Given inconsistencies in the past literature and to ensure replicability, we conducted within-sample replication.^44^ To this end, data were split randomly into discovery (50%) and replication (50%) sets using the *caret* package in R. Linear mixed-effects models were then run within the discovery and replication sets. Correction for multiple comparisons was performed within each of the 4 models (1 main effect model plus 3 interaction models) using a false discovery rate (FDR; 75 comparisons in each model). We further Bonferroni corrected the FDR *P* value to account for the 4 models (ie, FDR-corrected *P* < .0125). A finding was considered significant if it was significant at FDR-corrected *P* < .0125 in both the discovery and replication sets. We conducted 10-fold, within-sample split-half replication to ensure that results were not dependent on the specific way the data was split, and we only interpreted replicable findings (ie, those that were significant in at least ≥5 folds). We report the number of significant folds for all brain regions in eTables 2 and 3 in the Supplement. We covaried for age, sex, scanner type (but not acquisition site; details are provided in eMethods 5 in the Supplement), and total brain volume (for subcortical volume and surface area variables^45^) in analyses, given their potential role as confounders^45,46,47,48^ (a directed acyclic graph is provided in eMethods 6 in the Supplement). Individuals missing data for the dependent variables, independent variables, and/or covariates were automatically excluded from analyses. Analyses were performed using standardized values. In addition, family was modeled as a random effect. For future meta-analytic purposes, we also report the following: (1) results of associations of income-to-needs ratio, education, and neighborhood SES (in separate models) and brain structure (as preregistered) and (2) results for surface area without total brain volume as a covariate (eTables 5 and 6 in the Supplement, respectively). In sensitivity analyses, we report the following: (1) results covarying for race or ethnicity, (2) results covarying for mean cortical thickness, (3) results including site as a random effect, and (4) results including rescaled propensity scores in the model (eTables 4, 8, 9, and 10 in the Supplement, respectively). In addition to preregistered analyses, given the suggestion that interpretations of uncorrected regional differences are incomplete without examining global patterns across the brain,^49^ we conducted 3 additional tests to examine the association between the 3 SES indicators (in the same model) and total volume and surface area as well as mean thickness (eTable 7 in the Supplement).

## Results

### Demographic Information

The sample used in the main analysis comprised 8862 children aged 9 to 10 years, with a mean (SD) age of 119.1 (7.5) months. There were 4243 girls (47.9%) and 4619 boys (52.1%). Data on race or ethnicity were available for 8857 of 8862 participants: 173 (2.0%) were Asian, 1099 (12.4%) were Black or African American, 1688 (19.1%) were Hispanic, 4967 (56.1%) were White, and 930 (10.5%) reported multiple races or ethnicities. Descriptive statistics for SES variables are presented in Table 1.

**Table 1.** Participant Demographic Information^a^

Characteristic	Values
Total No. of participants	8862
Sex, No. (%)	
Boys	4619 (52.1)
Girls	4243 (47.9)
Age, mean (SD) [range], mo	119.1 (7.5) [107-133]
ADI, mean (SD) [range]	38.0 (26.5) [0-100]
Parental education, mean (SD) [range], y	15.3 (2.5) [3-22]
Income-to-needs ratio, mean (SD) [range]	3.7 (2.4) [0.07-12.36]
Race or ethnicity, No. (%)^b^	
Asian	173 (2.0)
Black or African American	1099 (12.4)
Hispanic	1688 (19.1)
White	4967 (56.1)
Multiracial/multiethnic	930 (10.5)

Abbreviation: ADI, area deprivation index.

^a^ For information on scanner manufacturer by site, see eTable 1 in the Supplement.

^b^ Data were available for 8857 participants.

### Neighborhood Disadvantage and Brain Structure

**Figure 1.**
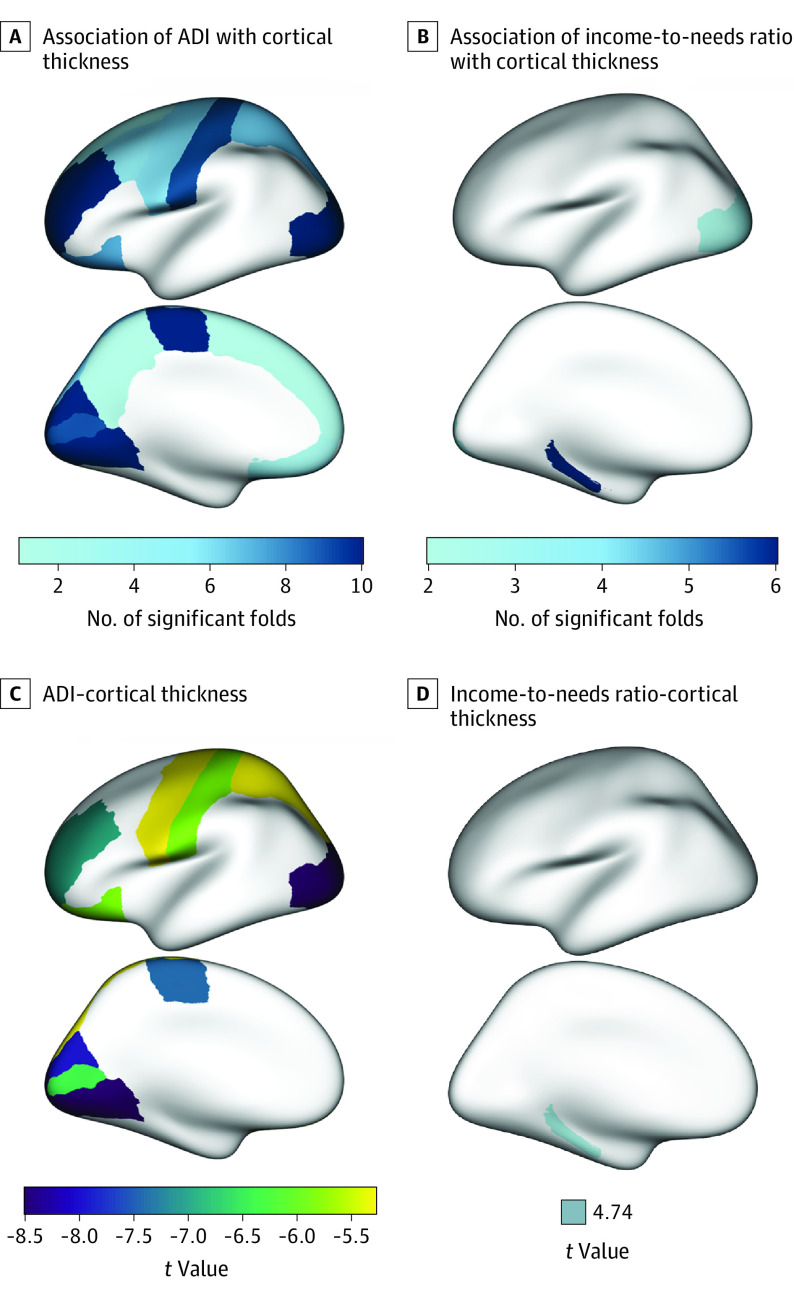
Independent Associations Between Socioeconomic Status Indicators and Brain Structure A and B, Number of significant folds for associations between cortical thickness and area deprivation index (ADI) (A) and income-to-needs ratio (B). C and D, *t*-Statistic values from linear mixed-effects models for associations between significant cortical thickness variables and ADI (C) and income-to-needs ratio (D). In addition to the thickness variables depicted, ADI was also associated with the superior temporal area in 1 fold.

Higher ADI was associated with lower thickness in a number of brain regions across the frontal, parietal, and occipital lobes (η^2^ = 0.004-0.009; Figure 1 and Table 2). Specifically, higher ADI was associated with reduced thickness in the cuneus (*B* [SE] = −0.099 [0.013]; *t* = −7.807; *P* < .001), lateral occipital (*B* [SE] = −0.088 [0.011]; *t* = −8.381; *P* < .001), lateral orbitofrontal (*B* [SE] = −0.072 [0.012]; *t* = −5.810; *P* < .001), lingual (*B* [SE] = −0.104 [0.012]; *t* = −8.495; *P* < .001), paracentral (*B* [SE] = −0.086 [0.012]; *t* = −7.068; *P* < .001), pericalcarine (*B* [SE] = −0.077 [0.012]; *t* = −6.199; *P* < .001), postcentral (*B* [SE] = −0.069 [0.012]; *t* = −5.961; *P* < .001), precentral (*B* [SE] = −0.059 [0.011]; *t* = −5.441; *P* < .001), rostral middle frontal (*B* [SE] = −0.076 [0.011]; *t* = −6.944; *P* < .001), superior parietal (*B* [SE] = −0.060 [0.011]; *t* = −5.286; *P* < .001).

**Table 2.** Model Output for Significant Models^a^

Cortical thickness variable	*B* (SE)	*t*	*P* value^b^	η^2^^c^
ADI				
Cuneus	−0.099 (0.013)	−7.807	6.62 × 10^−15^	0.008
Lateral occipital	−0.088 (0.011)	−8.381	6.16 × 10^−17^	0.009
Lateral orbitofrontal	−0.072 (0.012)	−5.810	6.49 × 10^−09^	0.004
Lingual	−0.104 (0.012)	−8.495	2.34 × 10^−17^	0.009
Paracentral	−0.086 (0.012)	−7.068	1.71 × 10^−12^	0.006
Pericalcarine	−0.077 (0.012)	−6.199	5.97 × 10^−10^	0.005
Postcentral	−0.069 (0.012)	−5.961	2.62 × 10^−09^	0.005
Precentral	−0.059 (0.011)	−5.441	5.45 × 10^−08^	0.004
Rostral middle frontal	−0.076 (0.011)	−6.944	4.11 × 10^−12^	0.006
Superior parietal	−0.060 (0.011)	−5.286	1.29 × 10^−07^	0.004
Income-to-needs ratio				
Insula	0.079 (0.014)	5.638	1.79 × 10^−8^	0.004
ADI × income-to-needs ratio				
Cuneus	0.068 (0.012)	5.715	1.13 × 10^−08^	0.004
Lateral occipital	0.074 (0.010)	7.523	5.93 × 10^−14^	0.007
Lateral orbitofrontal	0.066 (0.012)	5.658	1.59 × 10^−08^	0.004
Lingual	0.069 (0.012)	5.925	3.25 × 10^−09^	0.004
Pericalcarine	0.071 (0.012)	6.070	1.34 × 10^−09^	0.005
Insula	0.057 (0.012)	4.876	1.10 × 10^−06^	0.003
Educational attainment × income-to-needs ratio				
Cuneus	−0.067 (0.011)	−6.087	1.20 × 10^−09^	0.004
Lateral occipital	−0.057 (0.009)	−6.232	4.83 × 10^−10^	0.005
Lateral orbitofrontal	−0.050 (0.011)	−4.676	2.97 × 10^−06^	0.003
Lingual	−0.066 (0.011)	−6.135	8.91 × 10^−10^	0.005
Parahippocampal	−0.052 (0.011)	−4.734	2.23 × 10^−06^	0.003
Pericalcarine	−0.083 (0.011)	−7.696	1.56 × 10^−14^	0.007

Abbreviation: ADI, area deprivation index.

^a^ Model output was extracted using the whole sample. Results for main effects of ADI and income-to-needs ratio are from models that included all 3 socioeconomic status indicators.

^b^ Uncorrected *P* values are reported. See eTables 2 and 3 in the Supplement for model output for all other variables.

^c^ η^2^ was calculated using the effect size package in R (function t_to_eta2).

A higher income-to-needs ratio was associated with higher cortical thickness in the parahippocampal gyrus (*B* [SE] = 0.079 [0.014]; *t* = 5.638; *P* < .001; Figure 1 and Table 2). See Figure 1A and 1B and eTable 2 in the Supplement for the number of significant folds for each brain region (and for model output for all variables). There were no significant findings for cortical surface area or subcortical volume, and educational attainment was not robustly associated with the structure of any brain regions.

### Joint Associations Between SES Indicators and Brain Structure

**Figure 2.**
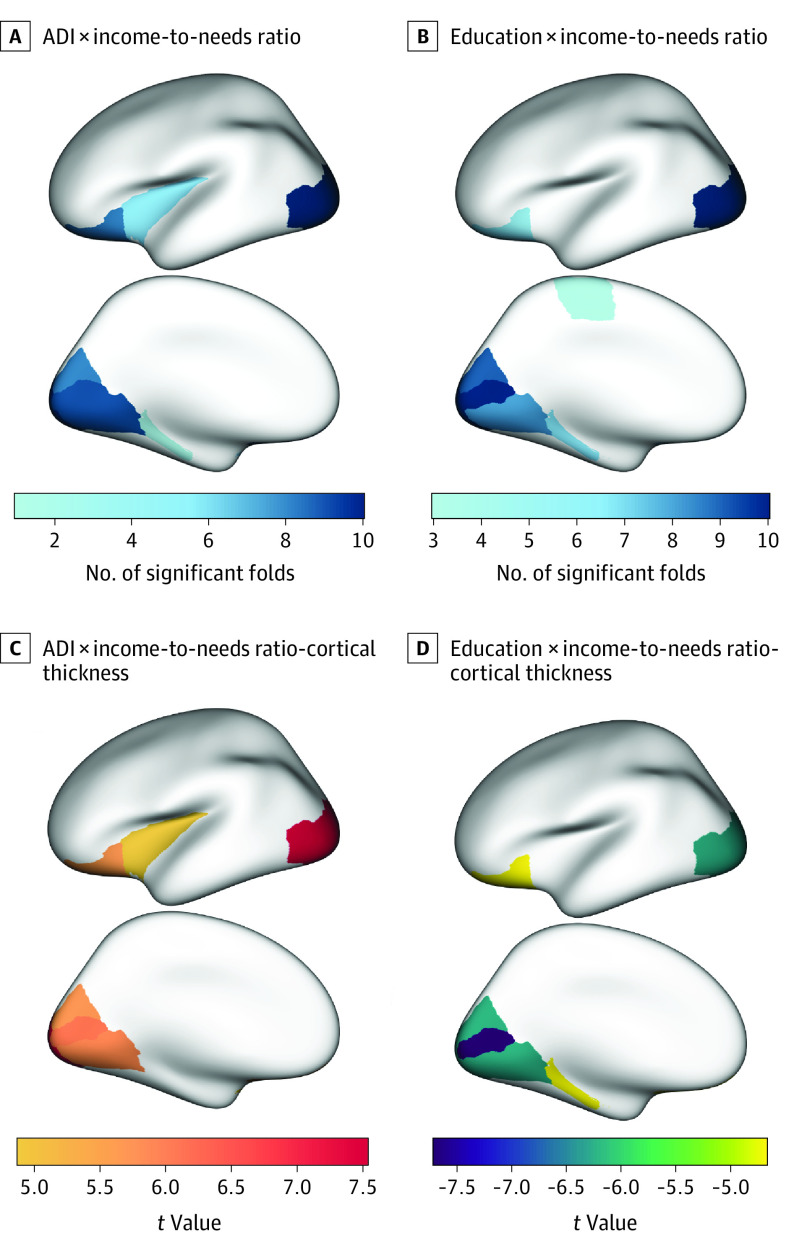
Interactive Associations Between Socioeconomic Status Indicators and Brain Structure A and B, Number of significant folds for associations between cortical thickness and area deprivation index (ADI) × income-to-needs ratio (A) and educational attainment × income-to-needs ratio (B). C and D, *t*-Statistic values from linear mixed-effects models for associations between cortical thickness and ADI × income-to-needs ratio (C) and educational attainment × income-to-needs ratio (D).

**Figure 3.**
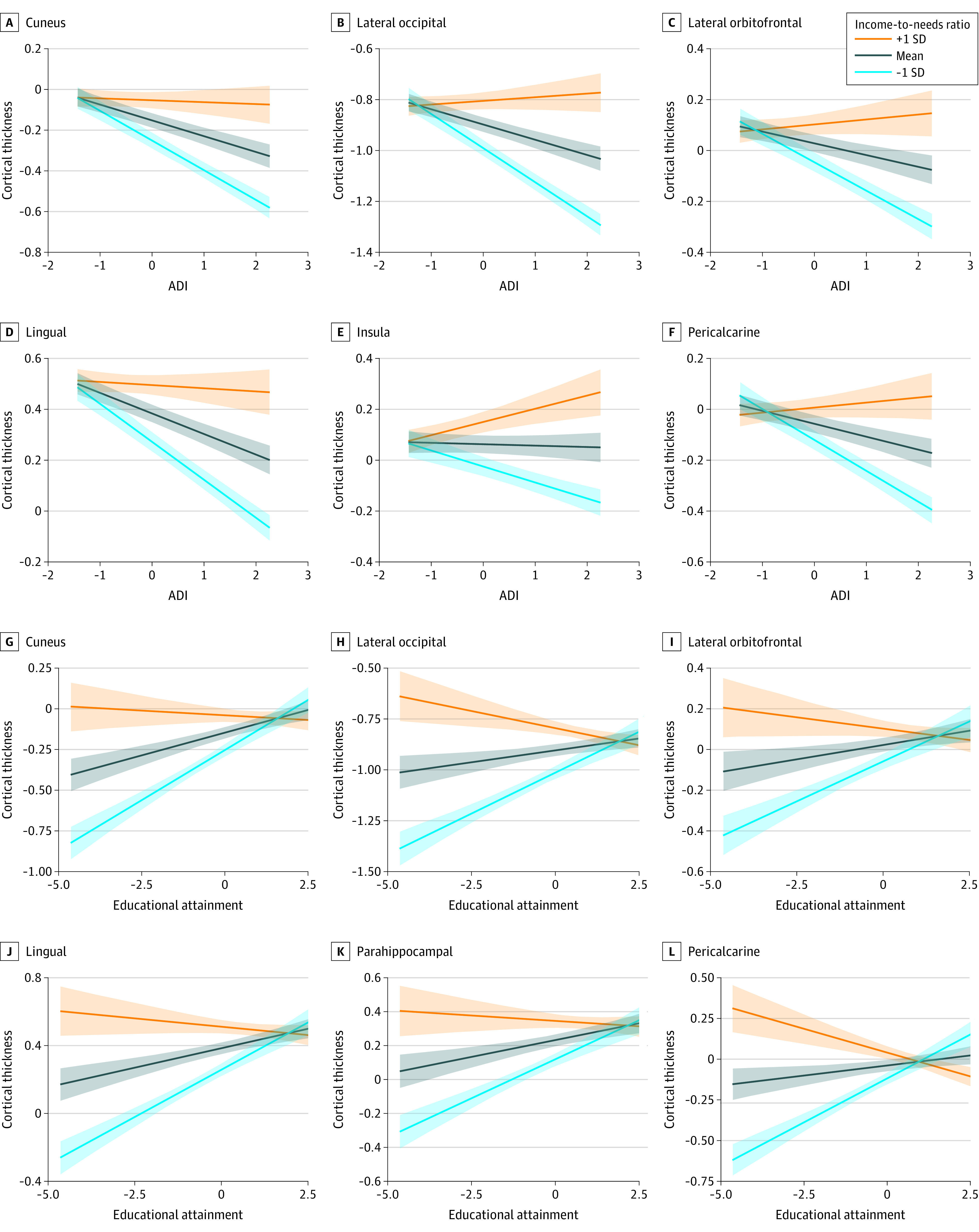
Associations Between Brain Structure and Neighborhood Disadvantage × Income-to-Needs Ratio and Education × Income-to-Needs Ratio A to F, Associations between area deprivation index (ADI) (x-axis) and cortical thickness (y-axis) are depicted at different income-to-needs ratios for the cuneus (A), lateral occipital (B), lateral orbitofrontal (C), lingual (D), insula (E), and pericalcarine (F) brain regions. G to L, Associations between educational attainment (x-axis) and cortical thickness (y-axis) are depicted at different income-to-needs ratios for the cuneus (G), lateral occipital (H), lateral orbitofrontal (I), lingual (J), parahippocampal (K), and pericalcarine (L) brain regions. Slopes represent mean (±1 SD) of income-to-needs ratios. Figures visualize standardized values.

We found a significant interaction between ADI and income-to-needs ratio for thickness in the cuneus (*B* [SE] = 0.068 [0.012]; *t* = 5.715; *P* < .001), lateral occipital (*B* [SE] = 0.074 [0.010]; *t* = 7.523; *P* < .001), lateral orbitofrontal (*B* [SE] = 0.066 [0.012]; *t* = 5.658; *P* < .001), lingual (*B* [SE] = 0.069 [0.012]; *t* = 5.925; *P* < .001), pericalcarine (*B* [SE] = 0.071 [0.012]; *t* = 6.070; *P* < .001), and insula (*B* [SE] = 0.057 [0.012]; *t* = 4.876; *P* < .001) (Figure 2). We also found a significant interaction between educational attainment and income-to-needs ratio for thickness of a number of the same regions, including the cuneus (*B* [SE] = −0.067 [0.011]; *t* = −6.087; *P* < .001), lateral occipital (*B* [SE] = −0.057 [0.009]; *t* = −6.232; *P* < .001), lateral orbitofrontal (*B* [SE] = −0.050 [0.011]; *t* = −4.676; *P* < .001), lingual (*B* [SE] = −0.066 [0.011]; *t* = −6.135; *P* < .001), parahippocampal (*B* [SE] = −0.052 [0.011]; *t* = −4.734; *P* < .001), and pericalcarine (*B* [SE] = −0.083 [0.011]; *t* = −7.696; *P* < .001) (Figure 2). Specifically, the negative association between ADI or low educational attainment and reduced cortical thickness was less pronounced in the presence of a high income-to-needs ratio for all variables (Figure 3). Model output is provided in Table 2; an illustration of the associations and the number of significant folds for each region are provided in Figure 3. There were no significant findings for cortical surface area or subcortical volume. The number of significant folds and model output for all variables are provided in eTable 3 in the Supplement.

## Discussion

The goal of this cross-sectional study was to examine the independent and joint associations between different indicators of SES and brain structure in a large sample of children aged 9 to 10 years. We found that neighborhood disadvantage was independently associated with cortical thickness of brain regions across frontal, parietal, and occipital lobes. Income-to-needs ratio was independently associated with parahippocampal thickness. In addition, we found that different SES indicators had synergistic associations, such that the association between neighborhood disadvantage or low educational attainment and reduced cortical thickness was less pronounced in the presence of high income-to-needs ratios. In general, the effect sizes were small, with the percentage of the variance in brain structure explained by the SES indices ranging from 0.003 to 0.009.

We found higher neighborhood disadvantage to be associated with reduced thickness of several brain regions across frontal (eg, dorsolateral prefrontal, and medial and lateral orbitofrontal cortices), parietal (eg, paracentral and postcentral cortices), and occipital (eg, cuneus, lingual, and lateral occipital cortices) lobes when accounting for household SES factors. Widespread associations between neighborhood disadvantage and brain structure (as well as associations above and beyond those of household SES) are consistent with previous work by us^29,50,51^ (approximately 8000 adolescents aged 9-10 years and 165 aged 12-19 years; 3 time points) and others^52,53,54^ (1012, 237, and 310 adolescents aged 8-22, 15, and 20 years, respectively) on brain structure and function. However, we did not find any associations between educational attainment and brain structure, very limited associations between income-to-needs ratio and brain structure, and no associations with surface area, in contrast to the associations observed in prior work (for a review, see Rakesh and Whittle^12^). For example, Noble et al^19^ reported extensive SES-associated alterations in surface area in 1099 youth aged 3 to 20 years. These differences could have arisen for multiple reasons. Specifically, most previous research investigating household SES has not included neighborhood disadvantage in their models and/or did not account for total brain volume in analyses of surface area.^15,19,55^ In support of these speculations, we report (1) associations between income-to-needs ratio or education and the surface area of several cortical regions when total brain volume was not included as a covariate and (2) several associations between education or income-to-needs ratio and brain structure in lower-order models with single SES indices (eTables 6 and 7 in the Supplement). Moreover, it is worth noting that we did observe associations with reduced total brain volume, total surface area, and mean thickness as a function of greater disadvantage for all 3 SES indicators (eTable 7 in the Supplement). As such, it is possible that associations between income or education and surface area of specific regions, above and beyond total brain-level associations, are not robust.

Furthermore, studies have reported SES-associated alterations in visual regions,^55,56,57,58,59,60^ which is consistent with our findings. Notwithstanding differences with prior literature, our finding of an association of neighborhood disadvantage with the thickness of frontoparietal and visual regions may also be consistent with recent suggestions that these regions play an important role in the association between SES and executive function.^61^ The model suggests that SES-associated variation in cognitive stimulation may scaffold the development of regions involved in cognitive processes via alterations in visual systems.^61^ Our findings extend this model and suggest that the neighborhood context may play a particularly salient role in shaping these systems. The important role that neighborhoods have been posited to play in shaping brain development is unsurprising given that neighborhoods likely affect child development in multiple ways, including exposure to stressful events and violence, access to community resources, quality of education and health care, and exposure to environmental contaminants.^62,63,64,65^ Furthermore, beyond being associated with structural community characteristics (eg, quality of education and health care available), neighborhood quality has also been linked to parenting practices. Studies have shown that disadvantaged neighborhoods are also associated with parenting behaviors, including the use of education-focused practices and parental warmth and monitoring.^66,67,68,69^ These observations could, in essence, mask effects of household SES and explain the independent associations between neighborhood disadvantage and brain structure.^51,54^ From the perspective of designing interventions, disentangling the extent to which these different aspects (both independent and overlapping) of the home and neighborhood environments influence child development is challenging, but it is a worthwhile avenue for future research.

Although we and others have reported associations between neighborhood disadvantage and brain development,^20,51,54^ it is important to understand why neighborhoods can affect child development. Several social, physical, and resource-based factors inherent to neighborhoods (eg, crime, cohesion among neighbors, presence of toxicants, access to libraries, school quality) change the nature of a child’s interaction with their environment,^70^ which could explain neighborhood-associated alterations in sociocognitive functions.^63,71,72^ For example, higher crime rates in poorer neighborhoods^31,32^ could contribute to increased stress,^33^ which is linked with executive function.^73,74^ Neuroimaging investigations have examined whether and which of these proximal factors may be associated with brain alterations. For example, pollution, toxicant exposure, and school environments have all been linked to brain structure and function.^75,76,77^ Future investigations of the mechanisms by which neighborhood disadvantage affects brain development using mediation frameworks and longitudinal designs are needed.

Moreover, we found that income-to-needs ratio moderated the association between neighborhood disadvantage or educational attainment and cortical thickness. The negative association between neighborhood disadvantage or low education and cortical thickness was reduced in the presence of a high income-to-needs ratio. Interestingly, the brain regions implicated across both models were similar. Specifically, the lateral orbitofrontal cortex, in addition to the medial and lateral occipital regions, was sensitive to the presence of a high income-to-needs ratio. Our findings indicate that regions of the occipital cortex are particularly sensitive to the different aspects of the socioeconomic context. Recent empirical evidence suggests that cognitive stimulation may serve as a mechanism linking SES to the functioning of visual regions.^78^ All 3 socioeconomic indicators are associated with cognitive stimulation in different ways. First, income-to-needs ratio is associated with access to complex learning material. Second, parent or caregiver educational attainment likely affects the linguistic environment and the strategies parents use to promote learning. Finally, neighborhood disadvantage is associated with access to community resources (eg, libraries). As a result, it is unsurprising that the presence of one source of cognitive stimulation can somewhat compensate for the absence of another.^61^ That said, work in this area is still in its nascent stages, and it is important to understand whether these interactions translate to behavioral outcomes in youth.

### Limitations

This study has some limitations. First, the study was cross-sectional, and brain outcomes were measured only once, at age 10 years. Therefore, we cannot comment on directionality or causality, and it is unknown whether the observed differences are transient or persistent. In addition, because of the observational nature of the study, it is likely there are other confounders we did not consider. Second, we did not test associations with mental health and behavior (eg, cognitive function); thus, we cannot comment on what the associations between SES and brain structure mean for children’s development more broadly. Future longitudinal work should test whether the brain alterations we observed mediate the association between different SES indicators and outcomes across a range of domains. Third, to ensure findings are driven by variation in the construct of interest rather than measurement artifacts, future work should use models that adjust for measurement biases.^79^ Fourth, the effect sizes in this study were small, but small effects can accumulate over time and be meaningful at the population level.^80^ However, the practical implications of these effects and how they evolve (ie, magnify or diminish) over time should be tested in future work. Fifth, in the absence of knowledge about how much time children spend in each household (eg, in a joint custody situation), we used ADI based on participants’ primary addresses. Future research with access to data on time spent at different addresses may benefit from using a weighted arithmetic mean of ADI. Finally, although the weighted estimates were highly similar, it must be noted that our findings may be specific to the ABCD sample and may not be generalizable to the overall US population.

## Conclusions

The findings of this cross-sectional study suggest that there are independent and joint associations between different indices of SES and brain structure. These findings highlight the importance of accounting for neighborhood disadvantage when examining associations between SES and child development. Our results also provide preliminary evidence for new targets that could form the basis of interventions and programs. For example, our findings suggest that programs aimed at reducing poverty (eg, through cash donations) may be beneficial in mitigating some of the negative effects of low education or neighborhood disadvantage on brain development. Our results also suggest that to minimize inequities among children and adolescents, preventative and/or intervention approaches should include an emphasis on improving neighborhood-level characteristics rather than family-level socioeconomic factors only. However, to design truly effective interventions, more research is needed (1) to disentangle the specific aspects of the neighborhood that may be most important for child development and (2) to understand whether brain changes associated with independent and joint SES factors are relevant for mental health and behavioral outcomes (eg, cognitive function).

## Notes

Supplement. **eMethods 1.** Distribution of Area Deprivation Index, Education, and Income-to-Needs Ratio **eMethods 2.** Pearson’s Bivariate Correlations Between Socioeconomic Status Variables **eMethods 3.** Income Bands **eMethods 4.** Participant Inclusion Flow Chart **eMethods 5.** Statistical Analyses **eMethods 6.** Directed Acyclic Graph **eTable 1.** Overlap Between Site and Scanner Manufacturer **eTable 2.** Model Output (Across the Whole Sample) for Area Deprivation Index, Education, and Income-to-Needs Ratio (Included in Models Simultaneously) **eTable 3.** Model Output (Across the Whole Sample) for Area Deprivation Index × Income-to-Needs Ratio, Area Deprivation Index × Education, and Income-to-Needs Ratio × Education Models **eTable 4.** Sensitivity Analysis: Model Output Controlling for Race or Ethnicity **eTable 5.** Model Output for Results for Individual Socioeconomic Status Variables **eTable 6.** Model Output for Results Without Controlling for Whole-Brain Volume for Surface Area Variables (When All 3 Socioeconomic Status Variables Are in the Same Model) **eTable 7.** Model Output for Whole-Brain Measures (When All 3 Socioeconomic Status Variables Are in the Same Model) **eTable 8.** Sensitivity Analysis: Model Output Controlling for Mean Thickness **eTable 9.** Sensitivity Analysis: Model Output Including Site as a Random Effect **eTable 10.** Sensitivity Analysis: Weighted Model Output (Accounting for Rescaled Propensity Scores)
eReferences

## References

[1] Forns J, Torrent M, Garcia-Esteban R, . Longitudinal association between early life socio-environmental factors and attention function at the age 11 years. Environ Res. 2012;117:54-59. doi:10.1016/j.envres.2012.04.007 22608140

[2] Ruijsbroek A, Wijga AH, Kerkhof M, Koppelman GH, Smit HA, Droomers M. The development of socio-economic health differences in childhood: results of the Dutch longitudinal PIAMA birth cohort. BMC Public Health. 2011;11:225. doi:10.1186/1471-2458-11-225 21486447 PMC3094243

[3] Koutra K, Chatzi L, Roumeliotaki T, . Socio-demographic determinants of infant neurodevelopment at 18 months of age: Mother-Child Cohort (Rhea Study) in Crete, Greece. Infant Behav Dev. 2012;35(1):48-59. doi:10.1016/j.infbeh.2011.09.005 22018719

[4] Packard CJ, Bezlyak V, McLean JS, . Early life socioeconomic adversity is associated in adult life with chronic inflammation, carotid atherosclerosis, poorer lung function and decreased cognitive performance: a cross-sectional, population-based study. BMC Public Health. 2011;11(1):42. doi:10.1186/1471-2458-11-42 21241479 PMC3032683

[5] Noble KG, McCandliss BD, Farah MJ. Socioeconomic gradients predict individual differences in neurocognitive abilities. Dev Sci. 2007;10(4):464-480. doi:10.1111/j.1467-7687.2007.00600.x 17552936

[6] Farah MJ, Shera DM, Savage JH, . Childhood poverty: specific associations with neurocognitive development. Brain Res. 2006;1110(1):166-174. doi:10.1016/j.brainres.2006.06.072 16879809

[7] McLaughlin KA, Breslau J, Green JG, . Childhood socio-economic status and the onset, persistence, and severity of DSM-IV mental disorders in a US national sample. Soc Sci Med. 2011;73(7):1088-1096. doi:10.1016/j.socscimed.2011.06.011 21820781 PMC3191493

[8] Farah MJ. Socioeconomic status and the brain: prospects for neuroscience-informed policy. Nat Rev Neurosci. 2018;19(7):428-438. doi:10.1038/s41583-018-0023-2 29867123

[9] Farah MJ. Child poverty and brain development. In: Sternberg R, Fiske S, Foss D, eds. Scientists Making a Difference: One Hundred Eminent Behavioral and Brain Scientists Talk About Their Most Important Contributions. Cambridge University Press; 2016:20-23.

[10] Hackman DA, Farah MJ, Meaney MJ. Socioeconomic status and the brain: mechanistic insights from human and animal research. Nat Rev Neurosci. 2010;11(9):651-659. doi:10.1038/nrn2897 20725096 PMC2950073

[11] Hackman DA, Farah MJ. Socioeconomic status and the developing brain. Trends Cogn Sci. 2009;13(2):65-73. doi:10.1016/j.tics.2008.11.003 19135405 PMC3575682

[12] Rakesh D, Whittle S. Socioeconomic status and the developing brain—a systematic review of neuroimaging findings in youth. Neurosci Biobehav Rev. 2021;130:379-407. doi:10.1016/j.neubiorev.2021.08.027 34474050

[13] Hanson JL, Chandra A, Wolfe BL, Pollak SD. Association between income and the hippocampus. PLoS One. 2011;6(5):e18712. doi:10.1371/journal.pone.0018712 21573231 PMC3087752

[14] Hanson JL, Nacewicz BM, Sutterer MJ, . Behavioral problems after early life stress: contributions of the hippocampus and amygdala. Biol Psychiatry. 2015;77(4):314-323. doi:10.1016/j.biopsych.2014.04.020 24993057 PMC4241384

[15] Lawson GM, Duda JT, Avants BB, Wu J, Farah MJ. Associations between children’s socioeconomic status and prefrontal cortical thickness. Dev Sci. 2013;16(5):641-652. doi:10.1111/desc.12096 24033570 PMC3775298

[16] Lichtin RD, Merz EC, He X, . Material hardship, prefrontal cortex-amygdala structure, and internalizing symptoms in children. Dev Psychobiol. 2021;63(2):364-377. doi:10.1002/dev.22020 32754912 PMC7858699

[17] Luby J, Belden A, Botteron K, . The effects of poverty on childhood brain development: the mediating effect of caregiving and stressful life events. JAMA Pediatr. 2013;167(12):1135-1142. doi:10.1001/jamapediatrics.2013.3139 24165922 PMC4001721

[18] Barch DM, Donohue MR, Elsayed NM, . Early childhood socioeconomic status and cognitive and adaptive outcomes at the transition to adulthood: the mediating role of gray matter development across five scan waves. Biol Psychiatry Cogn Neurosci Neuroimaging. 2022;7(1):34-44. doi:10.1016/j.bpsc.2021.07.002 34273554 PMC8917509

[19] Noble KG, Houston SM, Brito NH, . Family income, parental education and brain structure in children and adolescents. Nat Neurosci. 2015;18(5):773-778. doi:10.1038/nn.3983 25821911 PMC4414816

[20] Whittle S, Vijayakumar N, Simmons JG, . Role of positive parenting in the association between neighborhood social disadvantage and brain development across adolescence. JAMA Psychiatry. 2017;74(8):824-832. doi:10.1001/jamapsychiatry.2017.1558 28636697 PMC5710640

[21] Oakes JM, Rossi PH. The measurement of SES in health research: current practice and steps toward a new approach. Soc Sci Med. 2003;56(4):769-784. doi:10.1016/S0277-9536(02)00073-4 12560010

[22] Eamon MK. Social-demographic, school, neighborhood, and parenting influences on the academic achievement of Latino young adolescents. J Youth Adolesc. 2005;34(2):163-174. doi:10.1007/s10964-005-3214-x

[23] Sastry N, Pebley AR. Family and neighborhood sources of socioeconomic inequality in children’s achievement. Demography. 2010;47(3):777-800. doi:10.1353/dem.0.0114 20879688 PMC3000065

[24] Duncan GJ, Magnuson K. Socioeconomic status and cognitive functioning: moving from correlation to causation. Wiley Interdiscip Rev Cogn Sci. 2012;3(3):377-386. doi:10.1002/wcs.1176 26301469

[25] Taylor RL, Cooper SR, Jackson JJ, Barch DM. Assessment of neighborhood poverty, cognitive function, and prefrontal and hippocampal volumes in children. JAMA Netw Open. 2020;3(11):e2023774. doi:10.1001/jamanetworkopen.2020.23774 33141160 PMC7610187

[26] Dennis E, Manza P, Volkow ND. Socioeconomic status, BMI, and brain development in children. Transl Psychiatry. 2022;12(1):33. doi:10.1038/s41398-022-01779-3 35075111 PMC8786961

[27] Gordon RA, Savage C, Lahey BB, . Family and neighborhood income: additive and multiplicative associations with youths’ well-being. Soc Sci Res. 2003;32(2):191-219. doi:10.1016/S0049-089X(02)00047-9

[28] Kupersmidt JB, Griesler PC, DeRosier ME, Patterson CJ, Davis PW. Childhood aggression and peer relations in the context of family and neighborhood factors. Child Dev. 1995;66(2):360-375. doi:10.2307/1131583 7750371

[29] Rakesh D, Zalesky A, Whittle S. Similar but distinct—effects of different socioeconomic indicators on resting state functional connectivity: findings from the Adolescent Brain Cognitive Development (ABCD) Study. Dev Cogn Neurosci. 2021;51:101005. doi:10.1016/j.dcn.2021.101005 34419766 PMC8379618

[30] Perkins SC, Finegood ED, Swain JE. Poverty and language development: roles of parenting and stress. Innov Clin Neurosci. 2013;10(4):10-19. 23696954 PMC3659033

[31] Airaksinen J, Aaltonen M, Tarkiainen L, Martikainen P, Latvala A. Associations of neighborhood disadvantage and offender concentration with criminal behavior: between-within analysis in Finnish registry data. J Crim Justice. 2021;74:101813. doi:10.1016/j.jcrimjus.2021.101813

[32] Krivo LJ, Peterson RD. Extremely disadvantaged neighborhoods and urban crime. Soc Forces. 1996;75(2):619-650. doi:10.2307/2580416

[33] Theall KP, Shirtcliff EA, Dismukes AR, Wallace M, Drury SS. Association between neighborhood violence and biological stress in children. JAMA Pediatr. 2017;171(1):53-60. doi:10.1001/jamapediatrics.2016.2321 27842189 PMC5262476

[34] Lupien SJ, McEwen BS, Gunnar MR, Heim C. Effects of stress throughout the lifespan on the brain, behaviour and cognition. Nat Rev Neurosci. 2009;10(6):434-445. doi:10.1038/nrn2639 19401723

[35] McEwen BS, Nasca C, Gray JD. Stress effects on neuronal structure: hippocampus, amygdala, and prefrontal cortex. Neuropsychopharmacology. 2016;41(1):3-23. doi:10.1038/npp.2015.171 26076834 PMC4677120

[36] Adolescent Cognitive Brain Development Study. *ABCD Data Release 3.0*. 2021. Accessed August 25, 2021. https://abcdstudy.org

[37] Casey BJ, Cannonier T, Conley MI, ; ABCD Imaging Acquisition Workgroup. The Adolescent Brain Cognitive Development (ABCD) Study: imaging acquisition across 21 sites. Dev Cogn Neurosci. 2018;32:43-54. doi:10.1016/j.dcn.2018.03.001 29567376 PMC5999559

[38] Garavan H, Bartsch H, Conway K, . Recruiting the ABCD sample: design considerations and procedures. Dev Cogn Neurosci. 2018;32:16-22. doi:10.1016/j.dcn.2018.04.004 29703560 PMC6314286

[39] Kind AJH, Jencks S, Brock J, . Neighborhood socioeconomic disadvantage and 30-day rehospitalization: a retrospective cohort study. Ann Intern Med. 2014;161(11):765-774. doi:10.7326/M13-2946 25437404 PMC4251560

[40] Singh GK. Area deprivation and widening inequalities in US mortality, 1969-1998. Am J Public Health. 2003;93(7):1137-1143. doi:10.2105/AJPH.93.7.1137 12835199 PMC1447923

[41] Hagler DJ Jr, Hatton S, Cornejo MD, . Image processing and analysis methods for the Adolescent Brain Cognitive Development Study. Neuroimage. 2019;202:116091. doi:10.1016/j.neuroimage.2019.116091 31415884 PMC6981278

[42] Tisdall MD, Hess AT, Reuter M, Meintjes EM, Fischl B, van der Kouwe AJW. Volumetric navigators for prospective motion correction and selective reacquisition in neuroanatomical MRI. Magn Reson Med. 2012;68(2):389-399. doi:10.1002/mrm.23228 22213578 PMC3320676

[43] White N, Roddey C, Shankaranarayanan A, . PROMO: real-time prospective motion correction in MRI using image-based tracking. Magn Reson Med. 2010;63(1):91-105. doi:10.1002/mrm.22176 20027635 PMC2892665

[44] Saragosa-Harris NM, Chaku N, MacSweeney N, . A practical guide for researchers and reviewers using the ABCD Study and other large longitudinal datasets. Dev Cogn Neurosci. 2022;55:101115. doi:10.1016/j.dcn.2022.101115 35636343 PMC9156875

[45] Panizzon MS, Fennema-Notestine C, Eyler LT, . Distinct genetic influences on cortical surface area and cortical thickness. Cereb Cortex. 2009;19(11):2728-2735. doi:10.1093/cercor/bhp026 19299253 PMC2758684

[46] Tamnes CK, Bos MGN, van de Kamp FC, Peters S, Crone EA. Longitudinal development of hippocampal subregions from childhood to adulthood. Dev Cogn Neurosci. 2018;30(30):212-222. doi:10.1016/j.dcn.2018.03.009 29597156 PMC5945606

[47] Gogtay N, Giedd JN, Lusk L, . Dynamic mapping of human cortical development during childhood through early adulthood. Proc Natl Acad Sci U S A. 2004;101(21):8174-8179. doi:10.1073/pnas.0402680101 15148381 PMC419576

[48] Wierenga LM, Langen M, Oranje B, Durston S. Unique developmental trajectories of cortical thickness and surface area. Neuroimage. 2014;87:120-126. doi:10.1016/j.neuroimage.2013.11.010 24246495

[49] Hackman DA, Cserbik D, Chen J-CC, . Association of local variation in neighborhood disadvantage in metropolitan areas with youth neurocognition and brain structure. JAMA Pediatr. 2021;175(8):e210426. doi:10.1001/jamapediatrics.2021.0426 33938908 PMC8094040

[50] Rakesh D, Seguin C, Zalesky A, Cropley V, Whittle S. Associations between neighborhood disadvantage, resting-state functional connectivity, and behavior in the Adolescent Brain Cognitive Development Study: the moderating role of positive family and school environments. Biol Psychiatry Cogn Neurosci Neuroimaging. 2021;6(9):877-886. doi:10.1016/j.bpsc.2021.03.008 33771727

[51] Rakesh D, Cropley V, Zalesky A, Vijayakumar N, Allen NB, Whittle S. Neighborhood disadvantage and longitudinal brain-predicted-age trajectory during adolescence. Dev Cogn Neurosci. 2021;51:101002. doi:10.1016/j.dcn.2021.101002 34411954 PMC8377545

[52] Tooley UA, Mackey AP, Ciric R, . Associations between neighborhood SES and functional brain network development. Cereb Cortex. 2020;30(1):1-19. doi:10.1093/cercor/bhz066 31220218 PMC7029704

[53] Gur RE, Moore TM, Rosen AFG, . Burden of environmental adversity associated with psychopathology, maturation, and brain behavior parameters in youths. JAMA Psychiatry. 2019;76(9):966-975. doi:10.1001/jamapsychiatry.2019.0943 31141099 PMC6547104

[54] Gard AM, Maxwell AM, Shaw DS, . Beyond family-level adversities: exploring the developmental timing of neighborhood disadvantage effects on the brain. Dev Sci. 2021;24(1):e12985. doi:10.1111/desc.12985 32416027 PMC7669733

[55] Mackey AP, Finn AS, Leonard JA, . Neuroanatomical correlates of the income-achievement gap. Psychol Sci. 2015;26(6):925-933. doi:10.1177/0956797615572233 25896418 PMC4458190

[56] Rosen ML, Sheridan MA, Sambrook KA, Meltzoff AN, McLaughlin KA. Socioeconomic disparities in academic achievement: a multi-modal investigation of neural mechanisms in children and adolescents. Neuroimage. 2018;173:298-310. doi:10.1016/j.neuroimage.2018.02.043 29486324 PMC5944356

[57] Finn AS, Minas JE, Leonard JA, . Functional brain organization of working memory in adolescents varies in relation to family income and academic achievement. Dev Sci. 2017;20(5):1-15. doi:10.1111/desc.12450 27434857

[58] Romeo RR, Christodoulou JA, Halverson KK, . Socioeconomic status and reading disability: neuroanatomy and plasticity in response to intervention. Cereb Cortex. 2018;28(7):2297-2312. doi:10.1093/cercor/bhx131 28591795 PMC5998958

[59] King LS, Dennis EL, Humphreys KL, Thompson PM, Gotlib IH. Cross-sectional and longitudinal associations of family income-to-needs ratio with cortical and subcortical brain volume in adolescent boys and girls. Dev Cogn Neurosci. 2020;44:100796. doi:10.1016/j.dcn.2020.100796 32479375 PMC7525143

[60] Alnæs D, Kaufmann T, Marquand AF, Smith SM, Westlye LT. Patterns of sociocognitive stratification and perinatal risk in the child brain. Proc Natl Acad Sci U S A. 2020;117(22):12419-12427. doi:10.1073/pnas.2001517117 32409600 PMC7275714

[61] Rosen ML, Amso D, McLaughlin KA. The role of the visual association cortex in scaffolding prefrontal cortex development: a novel mechanism linking socioeconomic status and executive function. Dev Cogn Neurosci. 2019;39:100699. doi:10.1016/j.dcn.2019.100699 31446376 PMC6783336

[62] Hyde LW, Gard AM, Tomlinson RC, Burt SA, Mitchell C, Monk CS. An ecological approach to understanding the developing brain: examples linking poverty, parenting, neighborhoods, and the brain. Am Psychol. 2020;75(9):1245-1259. doi:10.1037/amp0000741 33382290 PMC8167378

[63] Evans GW. The environment of childhood poverty. Am Psychol. 2004;59(2):77-92. doi:10.1037/0003-066X.59.2.77 14992634

[64] Santiago CDC, Wadsworth ME, Stump J. Socioeconomic status, neighborhood disadvantage, and poverty-related stress: prospective effects on psychological syndromes among diverse low-income families. J Econ Psychol. 2011;32(2):218-230. doi:10.1016/j.joep.2009.10.008

[65] Wight RG, Aneshensel CS, Miller-Martinez D, . Urban neighborhood context, educational attainment, and cognitive function among older adults. Am J Epidemiol. 2006;163(12):1071-1078. doi:10.1093/aje/kwj176 16707655

[66] Burton LM, Robin RL. In the mix, yet on the margins: the place of families in urban neighborhood and child development research. J Marriage Fam. 2000;62(4):1114-1135. doi:10.1111/j.1741-3737.2000.01114.x

[67] Klebanov PK, Brooks-Gunn J, Duncan GJ. Does neighborhood and family poverty affect mothers’ parenting, mental health, and social support? J Marriage Fam. 1994;56(2):441-455. doi:10.2307/353111

[68] Shumow L, Lomax R. Parental efficacy: predictor of parenting behavior and adolescent outcomes. Parent Sci Pract. 2009;2(2):127-150. doi:10.1207/S15327922PAR0202_03

[69] Greenman E, Bodovski K, Reed K. Neighborhood characteristics, parental practices and children’s math achievement in elementary school. Soc Sci Res. 2011;40(5):1434-1444. doi:10.1016/j.ssresearch.2011.04.007 25125713 PMC4130168

[70] Hyde LW, Gard AM, Tomlinson RC, Suarez GL, Westerman HB. Parents, neighborhoods, and the developing brain. Child Dev Perspect. Published online April 20, 2022. doi:10.1111/cdep.12453

[71] Leventhal T, Brooks-Gunn J. The neighborhoods they live in: the effects of neighborhood residence on child and adolescent outcomes. Psychol Bull. 2000;126(2):309-337. doi:10.1037/0033-2909.126.2.309 10748645

[72] Slopen N, Heard-Garris N. Structural racism and pediatric health—a call for research to confront the origins of racial disparities in health. JAMA Pediatr. 2022;176(1):13-15. doi:10.1001/jamapediatrics.2021.3594 34633431

[73] Hennessey EP, Kepinska O, Haft SL, . Hair cortisol and dehydroepiandrosterone concentrations: associations with executive function in early childhood. Biol Psychol. 2020;155:107946. doi:10.1016/j.biopsycho.2020.107946 32805299 PMC7530148

[74] Hackman DA, Betancourt LM, Brodsky NL, Hurt H, Farah MJ. Neighborhood disadvantage and adolescent stress reactivity. Front Hum Neurosci. 2012;6(SEPTEMBER):277. 23091454 10.3389/fnhum.2012.00277 PMC3469875

[75] Miller JG, Dennis EL, Heft-Neal S, Jo B, Gotlib IH. Fine particulate air pollution, early life stress, and their interactive effects on adolescent structural brain development: a longitudinal tensor-based morphometry study. Cereb Cortex. 2022;32(10):2156-2169. doi:10.1093/cercor/bhab346 34607342 PMC9113318

[76] Pujol J, Martínez-Vilavella G, Macià D, . Traffic pollution exposure is associated with altered brain connectivity in school children. Neuroimage. 2016;129:175-184. doi:10.1016/j.neuroimage.2016.01.036 26825441

[77] Rakesh D, Zalesky A, Whittle S. The role of school environment in brain structure, connectivity, and mental health in children: a multimodal investigation. Biol Psychiatry Cogn Neurosci Neuroimaging. Published online February 2, 2022. doi:10.1016/j.bpsc.2022.01.006 35123109

[78] Rosen ML, Lurie LA, Sambrook KA, Meltzoff AN, McLaughlin KA. Neural mechanisms underlying the income-achievement gap: the role of the ventral visual stream. Dev Cogn Neurosci. 2021;52:101025. doi:10.1016/j.dcn.2021.101025 34700196 PMC8551593

[79] DeJoseph ML, Herzberg MP, Sifre RD, Berry D, Thomas KM. Measurement matters: An individual differences examination of family socioeconomic factors, latent dimensions of children’s experiences, and resting state functional brain connectivity in the ABCD sample. Dev Cogn Neurosci. 2022;53:101043. doi:10.1016/j.dcn.2021.101043 34915436 PMC8683693

[80] Funder DC, Ozer DJ. Evaluating effect size in psychological research: sense and nonsense. Adv Methods Pract Psychol Sci. 2019;2(2):156-168. doi:10.1177/2515245919847202

