# Supplementary material for: Assessment of Parent Income and Education, Neighborhood Disadvantage, and Child Brain Structure
Source: JAMA Netw Open. 2022 Aug 18;5(8):e2226208. doi: 10.1001/jamanetworkopen.2022.26208 (PMC9389347; doi:10.1001/jamanetworkopen.2022.26208)
Supplement: Supplement. — eMethods 1. Distribution of Area Deprivation Index, Education, and Income-to-Needs Ratio eMethods 2. Pearson’s Bivariate Correlations Between Socioeconomic Status Variables eMethods 3. Income Bands eMethods 4. Participant Inclusion Flow Chart eMethods 5. Statistical Analyses eMethods 6. Directed Acyclic Graph eTable 1. Overlap Between Site and Scanner Manufacturer eTable 2. Model Output (Across the Whole Sample) for Area Deprivation Index, Education, and Income-to-Needs Ratio (Included in Models Simultaneously) eTable 3. Model Output (Across the Whole Sample) for Area Deprivation Index × Income-to-Needs Ratio, Area Deprivation Index × Education, and Income-to-Needs Ratio × Education Models eTable 4. Sensitivity Analysis: Model Output Controlling for Race or Ethnicity eTable 5. Model Output for Results for Individual Socioeconomic Status Variables eTable 6. Model Output for Results Without Controlling for Whole-Brain Volume for Surface Area Variables (When All 3 Socioeconomic Status Variables Are in the Same Model) eTable 7. Model Output for Whole-Brain Measures (When All 3 Socioeconomic Status Variables Are in the Same Model) eTable 8. Sensitivity Analysis: Model Output Controlling for Mean Thickness eTable 9. Sensitivity Analysis: Model Output Including Site as a Random Effect eTable 10. Sensitivity Analysis: Weighted Model Output (Accounting for Rescaled Propensity Scores) eReferences [file jamanetwopen-e2226208-s001.pdf]

Supplementary Online Content

Rakesh D, Zalesky A, Whittle S. Assessment of parent income and education, neighborhood disadvantage, and child brain structure. *JAMA Netw Open*. 2022;5(8):e2226208. doi:10.1001/jamanetworkopen.2022.26208

eMethods 1. Distribution of Area Deprivation Index, Education, and Income-to-Needs Ratio
eMethods 2. Pearson's Bivariate Correlations Between Socioeconomic Status Variables
eMethods 3. Income Bands
eMethods 4. Participant Inclusion Flow Chart
eMethods 5. Statistical Analyses
eMethods 6. Directed Acyclic Graph
eTable 1. Overlap Between Site and Scanner Manufacturer
eTable 2. Model Output (Across the Whole Sample) for Area Deprivation Index, Education, and Income-to-Needs Ratio (Included in Models Simultaneously)
eTable 3. Model Output (Across the Whole Sample) for Area Deprivation Index × Income-to-Needs Ratio, Area Deprivation Index × Education, and Income-to-Needs Ratio × Education Models
eTable 4. Sensitivity Analysis: Model Output Controlling for Race or Ethnicity
eTable 5. Model Output for Results for Individual Socioeconomic Status Variables
eTable 6. Model Output for Results Without Controlling for Whole-Brain Volume for Surface Area Variables (When All 3 Socioeconomic Status Variables Are in the Same Model)
eTable 7. Model Output for Whole-Brain Measures (When All 3 Socioeconomic Status Variables Are in the Same Model)
eTable 8. Sensitivity Analysis: Model Output Controlling for Mean Thickness
eTable 9. Sensitivity Analysis: Model Output Including Site as a Random Effect
eTable 10. Sensitivity Analysis: Weighted Model Output (Accounting for Rescaled Propensity Scores)
eReferences

This supplementary material has been provided by the authors to give readers additional information about their work.

© 2022 Rakesh D et al. *JAMA Network Open*.

eMethods 1. Distribution of Area Deprivation Index, Education, and Income-to-Needs Ratio

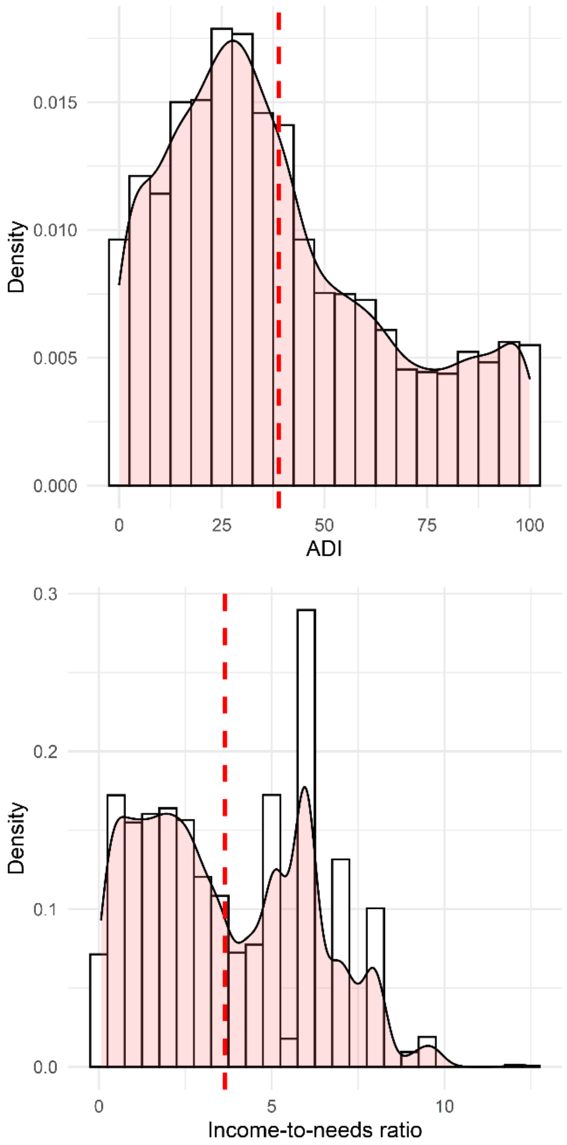

© 2022 Rakesh D et al. *JAMA Network Open*.

eMethods 2. Pearson's Bivariate Correlations Between Socioeconomic Status Variables

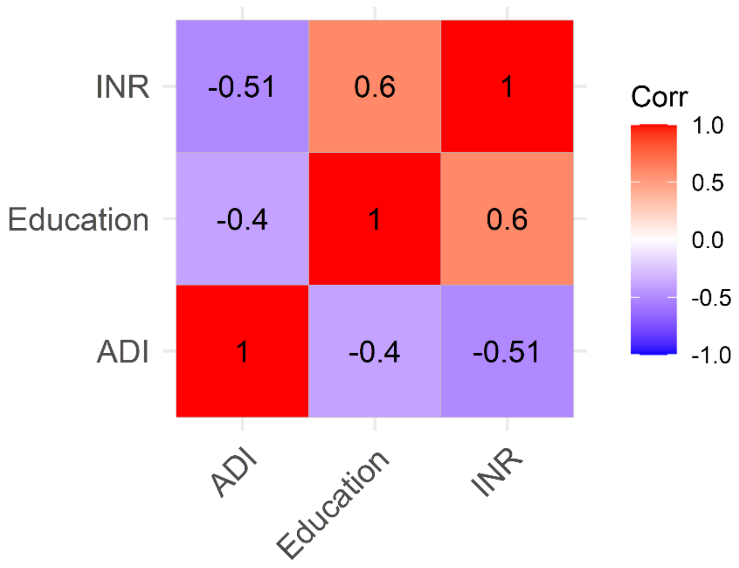

eMethods 3. Income Bands

1 = Less than $5,000

2 = $5,000 through $11,999

3 = $12,000 through $15,999

4 = $16,000 through $24,999

5 = $25,000 through $34,999

6 = $35,000 through $49,999

7 = $50,000 through $74,999

8 = $75,000 through $99,999

9 = $100,000 through $199,999

10 = $200,000 and greater

© 2022 Rakesh D et al. *JAMA Network Open*.

eMethods 4. Participant Inclusion Flow Chart

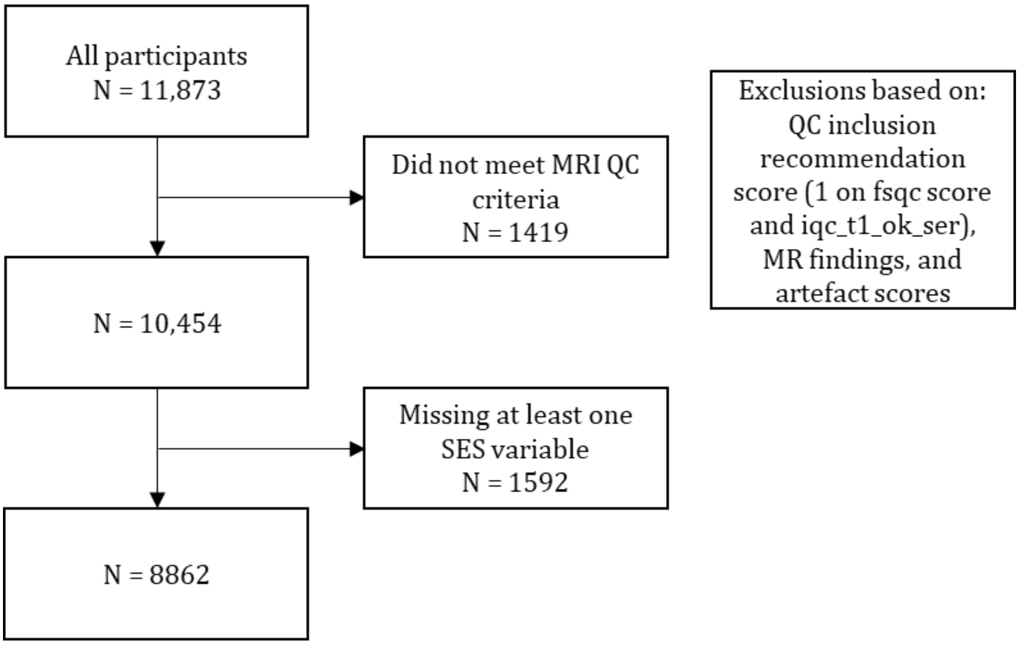

© 2022 Rakesh D et al. *JAMA Network Open*.

eMethods 5. Statistical Analyses

To examine the unique effects of the three SES indicators, and to account for both fixed (e.g., age) and random (e.g., multiple children from the same family) effects, we conducted linear mixed effects models (LMM; using the *lme4* package in R version 4.1.1) with ADI, parental educational attainment, and income-to-needs ratio as predictors (in the same model) and brain structure as the dependent variable (75 variables; in separate models). In three subsequent sets of models, we examined two-way interactions of 1) ADI x education, 2) ADI x income-to-needs, and 3) education x income-to-needs (lower order main effects were automatically included). Theories about interacting effects of SES indicators on child outcomes refer specifically to interactions between neighborhood and household indicators [1,2]; however, given that protective and additive effects are plausible for interactions between household SES indicators, we included the interaction between education and income-to-needs for completeness. We did not interpret main effects from models including interactions given that interpretation of such main effects would be overly complex.

Given inconsistencies in past literature and to ensure replicability we conducted within-sample replication[3]. To this end, data were split into discovery (50%) and replication (50%) sets randomly using the *caret* package in R. LMMs were then run within the discovery and replication sets. Correction for multiple comparisons was performed within each of the four models (one main effect model, plus three interaction models) using FDR (75 comparisons in each model). We further Bonferroni corrected the FDR p value to account for the four models (i.e., pFDR < 0.0125). A finding was considered as significant if it was significant at pFDR < 0.0125 in both the discovery and replication sets. We conducted ten folds of within-sample split-half replication to ensure that results were

© 2022 Rakesh D et al. *JAMA Network Open*.

not dependent on the specific way the data was split, and only interpreted replicable (i.e., those that replicated in at least ≥5 folds) findings. We report the number of significant folds for all regions in SI. We covaried for age, sex, scanner type (but not acquisition site; see SI for details), and total brain volume (for subcortical volume and surface area variables[4]) in analyses given their potential role as confounders[4–7]. Individuals missing data for the dependent variables, independent variables, and/or covariates were automatically excluded from models. In addition, family was modelled as a random effect. The inclusion of both site and scanner in the model sometimes led to model non-convergence within the discovery and replication sets (likely due to the smaller sample size) and made it challenging to compare results across folds. As such, we did not include site as a random effect in our final models, which is in line with recent work on the same sample[9]. Further, we also note that work on functional connectivity (using ABCD data) showed large systematic (i.e., reproducible) differences between scanners but only minimal effects for acquisition site[4]. In line with this, we found that the intra class coefficient indicated that site did not account for a significant amount of variance (range 0.01-0.09) when scanner, age, sex, and the three SES variables of interest were included in the same model. However, in order to demonstrate that there were minimal differences in estimates when site was included as a random effect, we conducted a sensitivity analysis and report the model output in eTable S9.

For future meta-analytic purposes, using the same within-sample split-half approach described above, we also report results of i) associations between income-to-needs/education/neighborhood SES (in separate models) and brain structure (as preregistered), and ii) results for surface area without total brain volume as a covariate. Results are available in this document. In sensitivity analyses, we report results i) covarying for race or ethnicity (eTable 4), ii) covarying for average cortical thickness

© 2022 Rakesh D et al. *JAMA Network Open*.

(eTable 8), iii) including site as a random effect (eTable 9), and iii) including rescaled propensity scores in the model. In addition to pre-registered analyses, given suggestion that interpretations of uncorrected regional differences are incomplete without examining global patterns across the brain[49], we conducted three additional tests to examine the association between the three SES indicators (in the same model) and total volume and surface area, and mean thickness (results in eTable 7).

eMethods 6. Directed Acyclic Graph

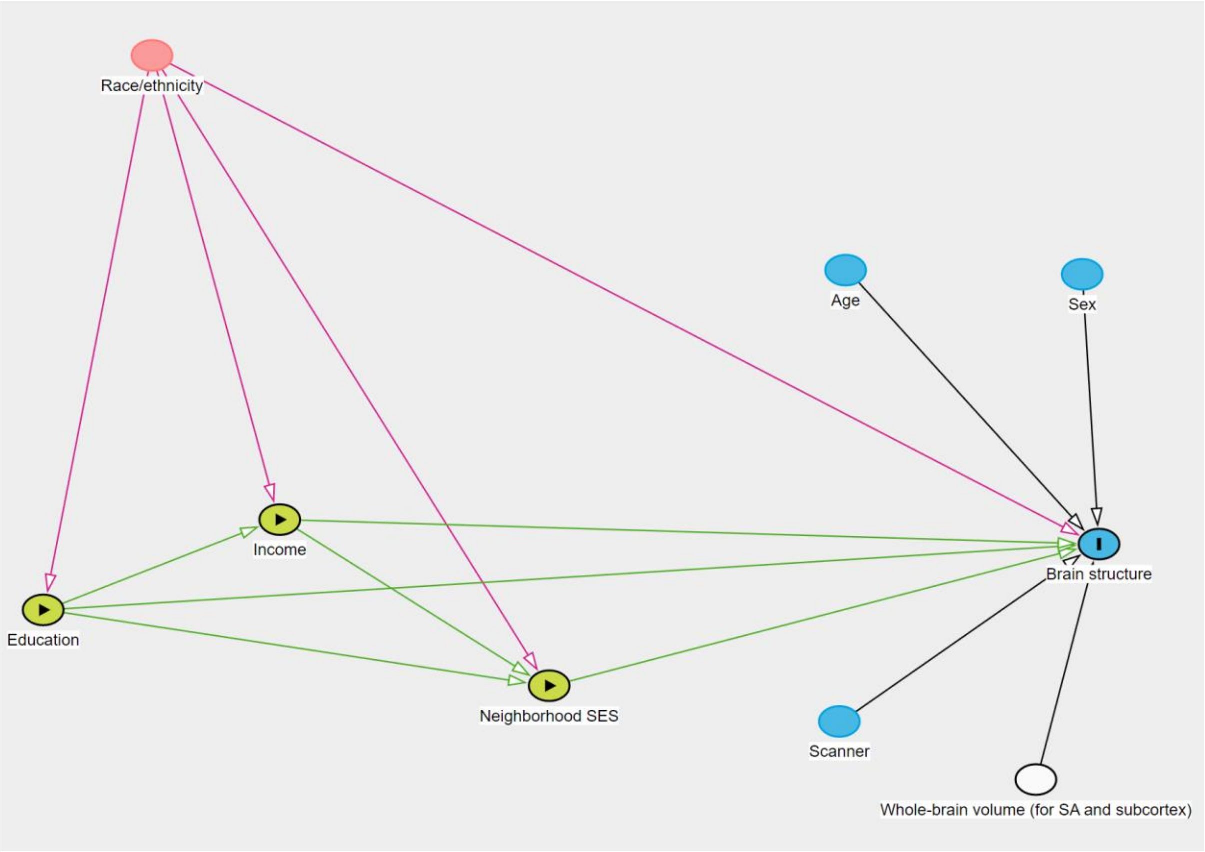

eTable 1. Overlap Between Site and Scanner Manufacturer

Scanner	site 01	site 02	site 03	site 04	site 05	site 06	site 07	site 08	site 09	site 10	site 11	site 12	site 13	site 14	site 15	site 16	site 17	site 18	site 19	site 20	site 21	site 22
GE_MEDICAL_SYSTEMS_DISCOVERY_MR750	0	0	0	603	0	0	0	291	1	630	0	0	567	0	0	0	0	323	0	0	0	27
Philips_Medical_Systems_Achieva_dStream	353	0	0	0	0	0	0	0	0	0	0	0	0	0	0	0	502	0	0	0	0	0
Philips_Medical_Systems_Ingenia	0	0	0	0	0	0	0	0	0	0	0	0	0	0	0	0	0	0	459	0	0	0
SIEMENS_Prisma	0	0	576	0	0	0	0	0	0	0	408	0	0	465	0	955	0	0	0	429	17	0
SIEMENS_Prisma_fit	0	505	0	0	350	535	295	1	397	0	0	540	0	85	379	0	0	0	0	208	517	0

© 2022 Rakesh D et al. *JAMA Network Open*.

eTable 2. Model Output (Across the Whole Sample) for Area Deprivation Index, Education, and Income-to-Needs Ratio (Included in Models Simultaneously)

	ADI						Education						INR					
	B	SE	T	P	Eta2	Folds	B	SE	T	P	Eta2	Folds	B	SE	T	P	Eta2	Folds
BANKS_OF_SUPERIOR_TEMPORAL_SULCUS_THICKNESS	-0.036	0.012	-3.051	2.29E-03	0.001	0	0.053	0.013	4.069	4.78E-05	0.002	0	0.036	0.013	2.742	6.11E-03	0.001	0
CAUDALANTERIORCINGULATE_THICKNESS	-0.020	0.013	-1.624	1.04E-01	0.000	0	-0.016	0.014	-1.150	2.50E-01	0.000	0	0.004	0.014	0.255	7.98E-01	0.000	0
CAUDALMIDDLEFRONTAL_THICKNESS	-0.056	0.012	-4.859	1.21E-06	0.003	4	0.000	0.013	-0.013	9.90E-01	0.000	0	0.008	0.013	0.653	5.13E-01	0.000	0
CUNEUS_THICKNESS	-0.099	0.013	-7.807	6.62E-15	0.008	10	0.056	0.014	4.068	4.78E-05	0.002	0	0.049	0.014	3.512	4.47E-04	0.002	0
ENTORHINAL_THICKNESS	-0.013	0.012	-1.058	2.90E-01	0.000	0	0.008	0.014	0.601	5.48E-01	0.000	0	0.022	0.014	1.551	1.21E-01	0.000	0
FUSIFORM_THICKNESS	-0.038	0.012	-3.157	1.60E-03	0.001	0	0.055	0.013	4.281	1.88E-05	0.002	0	0.047	0.013	3.563	3.69E-04	0.002	0
INFERIORPARIETAL_THICKNESS	-0.011	0.010	-1.071	2.84E-01	0.000	0	0.033	0.011	2.968	3.01E-03	0.001	0	0.020	0.011	1.745	8.11E-02	0.000	0
INFERIORTEMPORAL_THICKNESS	-0.003	0.012	-0.301	7.63E-01	0.000	0	0.048	0.013	3.843	1.23E-04	0.002	0	0.057	0.013	4.468	8.01E-06	0.003	0
ISTHMUSCINGULATE_THICKNESS	-0.006	0.013	-0.440	6.60E-01	0.000	0	0.010	0.014	0.698	4.85E-01	0.000	0	0.004	0.015	0.302	7.63E-01	0.000	0
LATERALOCCIPITAL_THICKNESS	-0.088	0.011	-8.381	6.16E-17	0.009	10	0.023	0.011	2.018	4.37E-02	0.001	0	0.059	0.012	5.024	5.18E-07	0.003	2
LATERALORBITOFRONTAL_THICKNESS	-0.072	0.012	-5.810	6.49E-09	0.004	7	0.026	0.013	1.903	5.71E-02	0.000	0	0.041	0.014	3.000	2.71E-03	0.001	0
LINGUAL_THICKNESS	-0.104	0.012	-8.495	2.34E-17	0.009	10	0.044	0.013	3.307	9.48E-04	0.001	0	0.068	0.014	4.996	5.98E-07	0.003	0
MEDIALORBITOFRONTAL_THICKNESS	-0.056	0.012	-4.582	4.67E-06	0.003	2	-0.007	0.013	-0.530	5.96E-01	0.000	0	0.006	0.014	0.415	6.78E-01	0.000	0
MIDDLETEMPORAL_THICKNESS	-0.022	0.010	-2.097	3.60E-02	0.001	0	0.043	0.011	3.789	1.52E-04	0.002	0	0.037	0.012	3.218	1.30E-03	0.001	0
PARAHIPPOCAMPAL_THICKNESS	-0.049	0.013	-3.844	1.22E-04	0.002	0	0.048	0.014	3.487	4.91E-04	0.002	0	0.079	0.014	5.638	1.79E-08	0.004	6
PARACENTRAL_THICKNESS	-0.086	0.012	-7.068	1.71E-12	0.006	10	0.038	0.013	2.919	3.52E-03	0.001	0	0.046	0.013	3.395	6.91E-04	0.001	0
PARSOPERCULARIS_THICKNESS	0.009	0.012	0.770	4.42E-01	0.000	0	0.018	0.013	1.417	1.57E-01	0.000	0	0.012	0.013	0.976	3.29E-01	0.000	0
PARSORBITALIS_THICKNESS	-0.009	0.012	-0.758	4.48E-01	0.000	0	0.025	0.013	1.876	6.06E-02	0.000	0	-0.001	0.014	-0.069	9.45E-01	0.000	0

© 2022 Rakesh D et al. *JAMA Network Open*.

**PARSTRIANGULARIS_THICKNESS**	-0.029	0.012	-2.529	1.15E-02	0.001	0	0.006	0.013	0.472	6.37E-01	0.000	0	0.009	0.013	0.701	4.83E-01	0.000	0
**PERICALCARINE_THICKNESS**	-0.077	0.012	-6.199	5.97E-10	0.005	9	0.032	0.014	2.336	1.95E-02	0.001	0	0.026	0.014	1.884	5.96E-02	0.000	0
**POSTCENTRAL_THICKNESS**	-0.069	0.012	-5.961	2.62E-09	0.005	9	0.035	0.013	2.811	4.96E-03	0.001	0	0.052	0.013	4.080	4.55E-05	0.002	0
**POSTERIORCINGULATE_THICKNESS**	0.009	0.012	0.687	4.92E-01	0.000	0	0.012	0.014	0.916	3.60E-01	0.000	0	0.017	0.014	1.193	2.33E-01	0.000	0
**PRECENTRAL_THICKNESS**	-0.059	0.011	-5.441	5.45E-08	0.004	5	0.032	0.012	2.685	7.27E-03	0.001	0	0.022	0.012	1.801	7.18E-02	0.000	0
**PRECUNEUS_THICKNESS**	-0.055	0.012	-4.515	6.42E-06	0.003	1	0.035	0.013	2.614	8.97E-03	0.001	0	0.037	0.014	2.706	6.81E-03	0.001	0
**ROSTRALANTERIORCINGULATE_THICKNESS**	-0.002	0.012	-0.145	8.84E-01	0.000	0	0.005	0.013	0.386	7.00E-01	0.000	0	-0.021	0.014	-1.545	1.22E-01	0.000	0
**ROSTRALMIDDLEFRONTAL_THICKNESS**	-0.076	0.011	-6.944	4.11E-12	0.006	10	0.019	0.012	1.591	1.12E-01	0.000	0	-0.001	0.012	-0.072	9.43E-01	0.000	0
**SUPERIORFRONTAL_THICKNESS**	-0.052	0.012	-4.501	6.87E-06	0.003	1	0.018	0.013	1.430	1.53E-01	0.000	0	-0.008	0.013	-0.658	5.10E-01	0.000	0
**SUPERIORPARIETAL_THICKNESS**	-0.060	0.011	-5.286	1.29E-07	0.004	6	0.032	0.012	2.565	1.03E-02	0.001	0	0.018	0.013	1.415	1.57E-01	0.000	0
**SUPERIORTEMPORAL_THICKNESS**	-0.005	0.011	-0.456	6.48E-01	0.000	0	0.060	0.012	4.913	9.16E-07	0.003	0	0.044	0.012	3.553	3.83E-04	0.002	0
**SUPRAMARGINAL_THICKNESS**	-0.040	0.009	-4.248	2.18E-05	0.002	0	0.019	0.010	1.815	6.95E-02	0.000	0	0.009	0.010	0.838	4.02E-01	0.000	0
**FRONTALPOLE_THICKNESS**	0.009	0.012	0.767	4.43E-01	0.000	0	0.031	0.013	2.319	2.04E-02	0.001	0	0.019	0.014	1.394	1.63E-01	0.000	0
**TEMPORALPOLE_THICKNESS**	0.001	0.012	0.085	9.32E-01	0.000	0	0.058	0.013	4.414	1.03E-05	0.003	0	0.027	0.013	2.034	4.20E-02	0.001	0
**TRANSVERSETEMPORAL_THICKNESS**	-0.024	0.012	-1.922	5.46E-02	0.000	0	0.052	0.014	3.828	1.30E-04	0.002	0	0.055	0.014	3.954	7.75E-05	0.002	0
**INSULA_THICKNESS**	-0.026	0.012	-2.050	4.04E-02	0.001	0	0.035	0.014	2.548	1.09E-02	0.001	0	0.052	0.014	3.741	1.84E-04	0.002	0
**BANKS_OF_SUPERIOR_TEMPORAL_SULCUS_AREA**	0.041	0.010	4.107	4.05E-05	0.002	0	0.019	0.011	1.729	8.39E-02	0.000	0	-0.007	0.011	-0.589	5.56E-01	0.000	0
**CAUDALANTERIORCINGULATE_AREA**	-0.013	0.011	-1.205	2.28E-01	0.000	0	-0.009	0.012	-0.818	4.13E-01	0.000	0	-0.020	0.012	-1.691	9.09E-02	0.000	0
**CAUDALMIDDLEFRONTAL_AREA**	0.001	0.009	0.149	8.82E-01	0.000	0	0.018	0.010	1.783	7.46E-02	0.000	0	0.012	0.011	1.128	2.59E-01	0.000	0
**CUNEUS_AREA**	-0.040	0.010	-3.839	1.24E-04	0.002	0	-0.036	0.011	-3.163	1.57E-03	0.001	0	-0.008	0.012	-0.691	4.89E-01	0.000	0
**ENTORHINAL_AREA**	-0.045	0.012	-3.912	9.23E-05	0.002	0	0.016	0.013	1.261	2.07E-01	0.000	0	0.014	0.013	1.129	2.59E-01	0.000	0
**FUSIFORM_AREA**	0.015	0.009	1.734	8.30E-02	0.000	0	0.016	0.009	1.732	8.33E-02	0.000	0	0.013	0.010	1.387	1.65E-01	0.000	0
**INFERIORPARIETAL_AREA**	0.006	0.009	0.708	4.79E-01	0.000	0	-0.005	0.009	-0.557	5.77E-01	0.000	0	-0.012	0.010	-1.276	2.02E-01	0.000	0

© 2022 Rakesh D et al. *JAMA Network Open*.

**INFERIORTEMPORAL_AREA**	-0.026	0.008	-3.159	1.59E-03	0.001	0	0.021	0.009	2.296	2.17E-02	0.001	0	-0.005	0.009	-0.577	5.64E-01	0.000	0
**ISTHMUSCINGULATE_AREA**	-0.009	0.010	-0.945	3.45E-01	0.000	0	-0.029	0.011	-2.731	6.32E-03	0.001	0	-0.032	0.011	-2.974	2.95E-03	0.001	0
**LATERALOCCIPITAL_AREA**	-0.003	0.009	-0.388	6.98E-01	0.000	0	0.005	0.010	0.483	6.29E-01	0.000	0	0.032	0.010	3.216	1.30E-03	0.001	0
**LATERALORBITOFRONTAL_AREA**	-0.001	0.008	-0.167	8.68E-01	0.000	0	-0.021	0.009	-2.262	2.37E-02	0.001	0	0.000	0.009	0.001	9.99E-01	0.000	0
**LINGUAL_AREA**	-0.004	0.011	-0.385	7.01E-01	0.000	0	0.001	0.012	0.075	9.40E-01	0.000	0	0.000	0.012	0.041	9.67E-01	0.000	0
**MEDIALORBITOFRONTAL_AREA**	0.022	0.009	2.555	1.06E-02	0.001	0	-0.026	0.009	-2.838	4.55E-03	0.001	0	0.006	0.009	0.635	5.25E-01	0.000	0
**MIDDLETEMPORAL_AREA**	-0.012	0.008	-1.457	1.45E-01	0.000	0	0.009	0.009	1.053	2.92E-01	0.000	0	0.001	0.009	0.104	9.17E-01	0.000	0
**PARAHIPPOCAMPAL_AREA**	0.028	0.011	2.614	8.98E-03	0.001	0	0.022	0.012	1.861	6.28E-02	0.000	0	-0.021	0.012	-1.754	7.94E-02	0.000	0
**PARACENTRAL_AREA**	0.027	0.011	2.536	1.12E-02	0.001	0	-0.020	0.012	-1.748	8.05E-02	0.000	0	0.009	0.012	0.792	4.29E-01	0.000	0
**PARSOPERCULARIS_AREA**	-0.022	0.010	-2.120	3.40E-02	0.001	0	-0.018	0.011	-1.578	1.15E-01	0.000	0	0.017	0.012	1.438	1.50E-01	0.000	0
**PARSORBITALIS_AREA**	-0.019	0.009	-2.094	3.63E-02	0.001	0	0.026	0.010	2.536	1.12E-02	0.001	0	0.011	0.010	1.059	2.89E-01	0.000	0
**PARSTRIANGULARIS_AREA**	0.016	0.011	1.451	1.47E-01	0.000	0	-0.002	0.012	-0.185	8.53E-01	0.000	0	-0.004	0.012	-0.358	7.20E-01	0.000	0
**PERICALCARINE_AREA**	-0.021	0.012	-1.756	7.92E-02	0.000	0	-0.003	0.013	-0.202	8.40E-01	0.000	0	-0.024	0.013	-1.850	6.44E-02	0.000	0
**POSTCENTRAL_AREA**	-0.009	0.008	-1.050	2.94E-01	0.000	0	-0.004	0.009	-0.415	6.78E-01	0.000	0	-0.005	0.009	-0.588	5.56E-01	0.000	0
**POSTERIORCINGULATE_AREA**	-0.022	0.010	-2.280	2.26E-02	0.001	0	-0.032	0.011	-3.040	2.37E-03	0.001	0	-0.020	0.011	-1.806	7.09E-02	0.000	0
**PRECENTRAL_AREA**	-0.023	0.008	-2.729	6.37E-03	0.001	0	-0.001	0.009	-0.118	9.06E-01	0.000	0	0.011	0.009	1.234	2.17E-01	0.000	0
**PRECUNEUS_AREA**	-0.026	0.008	-3.061	2.22E-03	0.001	0	-0.014	0.009	-1.462	1.44E-01	0.000	0	0.003	0.009	0.300	7.64E-01	0.000	0
**ROSTRALANTERIORCINGULATE_AREA**	0.009	0.009	1.024	3.06E-01	0.000	0	-0.016	0.010	-1.571	1.16E-01	0.000	0	-0.004	0.010	-0.429	6.68E-01	0.000	0
**ROSTRALMIDDLEFRONTAL_AREA**	0.011	0.008	1.342	1.80E-01	0.000	0	0.030	0.009	3.524	4.27E-04	0.002	0	-0.012	0.009	-1.387	1.66E-01	0.000	0
**SUPERIORFRONTAL_AREA**	0.014	0.007	2.017	4.38E-02	0.001	0	0.007	0.008	0.925	3.55E-01	0.000	0	0.008	0.008	1.026	3.05E-01	0.000	0
**SUPERIORPARIETAL_AREA**	-0.028	0.009	-3.091	2.00E-03	0.001	0	-0.016	0.010	-1.599	1.10E-01	0.000	0	0.005	0.010	0.526	5.99E-01	0.000	0
**SUPERIORTEMPORAL_AREA**	0.035	0.008	4.337	1.46E-05	0.002	1	0.023	0.009	2.591	9.60E-03	0.001	0	-0.003	0.009	-0.385	7.00E-01	0.000	0
**SUPRAMARGINAL_AREA**	-0.001	0.008	-0.131	8.96E-01	0.000	0	0.001	0.009	0.107	9.15E-01	0.000	0	-0.003	0.009	-0.367	7.14E-01	0.000	0
**FRONTALPOLE_AREA**	-0.021	0.011	-1.934	5.31E-02	0.000	0	0.030	0.012	2.498	1.25E-02	0.001	0	0.011	0.012	0.931	3.52E-01	0.000	0
**TEMPORALPOLE_AREA**	0.016	0.011	1.437	1.51E-01	0.000	0	0.010	0.012	0.840	4.01E-01	0.000	0	-0.008	0.012	-0.667	5.05E-01	0.000	0

© 2022 Rakesh D et al. *JAMA Network Open*.

**TRANSVERSETEMPORAL_AREA**	0.030	0.011	2.787	5.33E-03	0.001	0	-0.005	0.012	-0.454	6.50E-01	0.000	0	0.009	0.012	0.718	4.73E-01	0.000	0
**INSULA_AREA**	0.018	0.009	2.052	4.02E-02	0.001	0	-0.012	0.010	-1.237	2.16E-01	0.000	0	-0.031	0.010	-3.183	1.46E-03	0.001	0
**THALAMUS_SUB**	0.001	0.008	0.156	8.76E-01	0.000	0	-0.028	0.009	-3.272	1.07E-03	0.001	0	-0.019	0.009	-2.193	2.84E-02	0.001	0
**CAUDATE_SUB**	-0.012	0.011	-1.095	2.74E-01	0.000	0	0.034	0.012	2.872	4.09E-03	0.001	0	-0.019	0.012	-1.535	1.25E-01	0.000	0
**PUTAMEN_SUB**	-0.023	0.010	-2.198	2.80E-02	0.001	0	0.011	0.011	0.946	3.44E-01	0.000	0	-0.005	0.012	-0.406	6.85E-01	0.000	0
**PALLIDUM_SUB**	0.014	0.011	1.295	1.95E-01	0.000	0	-0.005	0.011	-0.411	6.81E-01	0.000	0	0.004	0.012	0.347	7.28E-01	0.000	0
**HIPPOCAMPUS_SUB**	-0.012	0.009	-1.218	2.23E-01	0.000	0	0.017	0.010	1.657	9.75E-02	0.000	0	0.010	0.011	0.987	3.24E-01	0.000	0
**AMYGDALA_SUB**	-0.020	0.009	-2.188	2.87E-02	0.001	0	-0.009	0.010	-0.892	3.72E-01	0.000	0	0.017	0.010	1.673	9.44E-02	0.000	0
**ACCUMBENS_SUB**	0.003	0.010	0.279	7.80E-01	0.000	0	-0.016	0.011	-1.512	1.31E-01	0.000	0	-0.016	0.011	-1.440	1.50E-01	0.000	0

Note: Table reports uncorrected p values. Effect size (eta^2) was computed using the t_to_eta2 function from the *effectsizes* package in R. Folds represents the number of folds the variable was significant in across discovery and replication samples at *p*FDR < 0.0125. Model equations included fixed effects ADI, education, income-to-needs, age, sex, scanner type, and whole-brain volume (for surface area and subcortical volume variables), and the random effect of family.

© 2022 Rakesh D et al. *JAMA Network Open*.

eTable 3. Model Output (Across the Whole Sample) for Area Deprivation Index × Income-to-Needs Ratio, Area Deprivation Index × Education, and Income-to-Needs Ratio × Education Models

	ADI*INR						ADI*Education						INR*Education					
	B	SE	T	P	Eta2	Folds	B	SE	T	P	Eta2	Folds	B	SE	T	P	Eta2	Folds
BANKS_OF_SUPERIOR_TEMPORAL_SULCUS_THICKNESS	0.040	0.011	3.585	3.39E-04	0.002	0	0.006	0.010	0.645	5.19E-01	0.000	0	-0.025	0.010	-2.373	1.77E-02	0.001	0
CAUDALANTERIORCINGULATE_THICKNESS	0.019	0.012	1.622	1.05E-01	0.000	0	0.001	0.010	0.050	9.60E-01	0.000	0	-0.019	0.011	-1.780	7.52E-02	0.000	0
CAUDALMIDDLEFRONTAL_THICKNESS	0.007	0.011	0.684	4.94E-01	0.000	0	-0.011	0.010	-1.138	2.55E-01	0.000	0	-0.045	0.010	-4.436	9.30E-06	0.002	0
CUNEUS_THICKNESS	0.068	0.012	5.715	1.13E-08	0.004	8	0.014	0.010	1.304	1.92E-01	0.000	0	-0.067	0.011	-6.087	1.20E-09	0.004	9
ENTORHINAL_THICKNESS	0.009	0.012	0.783	4.33E-01	0.000	0	0.012	0.010	1.111	2.67E-01	0.000	0	-0.009	0.011	-0.858	3.91E-01	0.000	0
FUSIFORM_THICKNESS	0.037	0.011	3.306	9.50E-04	0.001	0	-0.003	0.010	-0.300	7.64E-01	0.000	0	-0.029	0.010	-2.808	5.00E-03	0.001	0
INFERIORPARIETAL_THICKNESS	0.029	0.010	3.015	2.58E-03	0.001	0	0.001	0.008	0.144	8.85E-01	0.000	0	-0.024	0.009	-2.701	6.92E-03	0.001	0
INFERIORTEMPORAL_THICKNESS	0.016	0.011	1.447	1.48E-01	0.000	0	-0.016	0.010	-1.712	8.69E-02	0.000	0	-0.020	0.010	-1.979	4.79E-02	0.000	0
ISTHMUSCINGULATE_THICKNESS	0.009	0.012	0.725	4.68E-01	0.000	0	-0.006	0.011	-0.563	5.73E-01	0.000	0	-0.017	0.011	-1.524	1.28E-01	0.000	0
LATERALOCCIPITAL_THICKNESS	0.074	0.010	7.523	5.93E-14	0.007	10	0.020	0.009	2.257	2.40E-02	0.001	0	-0.057	0.009	-6.232	4.83E-10	0.005	10
LATERALORBITOFRONTAL_THICKNESS	0.066	0.012	5.658	1.59E-08	0.004	8	0.026	0.010	2.491	1.27E-02	0.001	0	-0.050	0.011	-4.676	2.97E-06	0.003	5
LINGUAL_THICKNESS	0.069	0.012	5.925	3.25E-09	0.004	9	0.017	0.010	1.614	1.07E-01	0.000	0	-0.066	0.011	-6.135	8.91E-10	0.005	8
MEDIALORBITOFRONTAL_THICKNESS	0.022	0.012	1.875	6.09E-02	0.000	0	-0.005	0.010	-0.516	6.06E-01	0.000	0	-0.025	0.011	-2.329	1.99E-02	0.001	0
MIDDLETEMPORAL_THICKNESS	0.005	0.010	0.481	6.31E-01	0.000	0	-0.013	0.009	-1.512	1.31E-01	0.000	0	-0.023	0.009	-2.599	9.37E-03	0.001	0
PARAHIPPOCAMPAL_THICKNESS	0.054	0.012	4.539	5.73E-06	0.003	1	0.032	0.011	3.077	2.10E-03	0.001	0	-0.052	0.011	-4.734	2.23E-06	0.003	6
PARACENTRAL_THICKNESS	0.053	0.011	4.636	3.62E-06	0.003	0	0.005	0.010	0.507	6.12E-01	0.000	0	-0.054	0.011	-5.151	2.66E-07	0.003	3
PARSOPERCULARIS_THICKNESS	0.011	0.011	1.018	3.09E-01	0.000	0	-0.003	0.010	-0.288	7.73E-01	0.000	0	-0.038	0.010	-3.845	1.21E-04	0.002	0
PARSORBITALIS_THICKNESS	0.013	0.012	1.122	2.62E-01	0.000	0	0.003	0.010	0.316	7.52E-01	0.000	0	-0.028	0.011	-2.634	8.46E-03	0.001	0

© 2022 Rakesh D et al. *JAMA Network Open*.

PARSTRIANGULARIS_THICKNESS	0.002	0.011	0.208	8.35E-01	0.000	0	-0.015	0.010	-1.540	1.24E-01	0.000	0	-0.017	0.010	-1.673	9.44E-02	0.000	0
PERICALCARINE_THICKNESS	0.071	0.012	6.070	1.34E-09	0.005	9	0.030	0.010	2.930	3.40E-03	0.001	0	-0.083	0.011	-7.696	1.56E-14	0.007	10
POSTCENTRAL_THICKNESS	0.033	0.011	3.056	2.25E-03	0.001	0	-0.013	0.010	-1.395	1.63E-01	0.000	0	-0.019	0.010	-1.910	5.62E-02	0.000	0
POSTERIORCINGULATE_THICKNESS	0.009	0.012	0.739	4.60E-01	0.000	0	-0.002	0.010	-0.148	8.82E-01	0.000	0	-0.018	0.011	-1.671	9.48E-02	0.000	0
PRECENTRAL_THICKNESS	0.041	0.010	4.037	5.46E-05	0.002	0	0.003	0.009	0.293	7.69E-01	0.000	0	-0.039	0.009	-4.103	4.12E-05	0.002	0
PRECUNEUS_THICKNESS	0.045	0.011	3.946	8.02E-05	0.002	0	0.004	0.010	0.445	6.56E-01	0.000	0	-0.043	0.011	-4.100	4.17E-05	0.002	0
ROSTRALANTERIORCINGULATE_THICKNESS	0.034	0.012	2.882	3.96E-03	0.001	0	0.006	0.010	0.561	5.75E-01	0.000	0	-0.016	0.011	-1.486	1.37E-01	0.000	0
ROSTRALMIDDLEFRONTAL_THICKNESS	0.014	0.010	1.355	1.75E-01	0.000	0	-0.011	0.009	-1.194	2.32E-01	0.000	0	-0.036	0.010	-3.753	1.76E-04	0.002	0
SUPERIORFRONTAL_THICKNESS	0.006	0.011	0.563	5.74E-01	0.000	0	-0.010	0.010	-0.985	3.24E-01	0.000	0	-0.036	0.010	-3.607	3.12E-04	0.002	0
SUPERIORPARIETAL_THICKNESS	0.041	0.011	3.796	1.48E-04	0.002	0	-0.002	0.009	-0.217	8.28E-01	0.000	0	-0.026	0.010	-2.614	8.96E-03	0.001	0
SUPERIORTEMPORAL_THICKNESS	0.005	0.011	0.452	6.52E-01	0.000	0	-0.008	0.009	-0.835	4.04E-01	0.000	0	-0.017	0.010	-1.758	7.88E-02	0.000	0
SUPRAMARGINAL_THICKNESS	0.022	0.009	2.526	1.16E-02	0.001	0	0.002	0.008	0.258	7.96E-01	0.000	0	-0.017	0.008	-2.099	3.58E-02	0.001	0
FRONTALPOLE_THICKNESS	-0.005	0.012	-0.425	6.71E-01	0.000	0	-0.021	0.010	-2.042	4.12E-02	0.000	0	-0.005	0.011	-0.467	6.40E-01	0.000	0
TEMPORALPOLE_THICKNESS	0.003	0.011	0.306	7.59E-01	0.000	0	-0.007	0.010	-0.721	4.71E-01	0.000	0	-0.001	0.010	-0.134	8.93E-01	0.000	0
TRANSVERSETEMPORAL_THICKNESS	0.034	0.012	2.928	3.42E-03	0.001	0	-0.003	0.010	-0.248	8.04E-01	0.000	0	-0.035	0.011	-3.210	1.33E-03	0.001	0
INSULA_THICKNESS	0.057	0.012	4.876	1.10E-06	0.003	5	0.018	0.010	1.778	7.55E-02	0.000	0	-0.036	0.011	-3.329	8.77E-04	0.001	0
BANKS_OF_SUPERIOR_TEMPORAL_SULCUS_AREA	-0.021	0.010	-2.165	3.04E-02	0.001	0	-0.016	0.008	-1.895	5.81E-02	0.000	0	0.023	0.009	2.607	9.16E-03	0.001	0
CAUDALANTERIORCINGULATE_AREA	-0.002	0.010	-0.220	8.26E-01	0.000	0	0.014	0.009	1.562	1.18E-01	0.000	0	0.004	0.009	0.400	6.89E-01	0.000	0
CAUDALMIDDLEFRONTAL_AREA	0.021	0.009	2.383	1.72E-02	0.001	0	0.018	0.008	2.312	2.08E-02	0.001	0	-0.022	0.008	-2.717	6.60E-03	0.001	0
CUNEUS_AREA	-0.011	0.010	-1.134	2.57E-01	0.000	0	0.001	0.009	0.142	8.87E-01	0.000	0	0.018	0.009	1.965	4.95E-02	0.000	0
ENTORHINAL_AREA	0.028	0.011	2.570	1.02E-02	0.001	0	0.010	0.010	1.021	3.08E-01	0.000	0	-0.013	0.010	-1.259	2.08E-01	0.000	0
FUSIFORM_AREA	-0.009	0.008	-1.084	2.78E-01	0.000	0	-0.008	0.007	-1.061	2.89E-01	0.000	0	0.014	0.007	1.844	6.53E-02	0.000	0
INFERIORPARIETAL_AREA	-0.016	0.008	-1.957	5.03E-02	0.000	0	-0.006	0.007	-0.905	3.65E-01	0.000	0	0.025	0.007	3.282	1.04E-03	0.001	0

© 2022 Rakesh D et al. *JAMA Network Open*.

**INFERIORTEMPORAL_AREA**	0.013	0.008	1.642	1.01E-01	0.000	0	0.011	0.007	1.592	1.11E-01	0.000	0	-0.002	0.007	-0.249	8.03E-01	0.000	0
**ISTHMUSCINGULATE_AREA**	-0.005	0.009	-0.588	5.56E-01	0.000	0	0.010	0.008	1.298	1.94E-01	0.000	0	0.010	0.008	1.129	2.59E-01	0.000	0
**LATERALOCCIPITAL_AREA**	0.011	0.008	1.265	2.06E-01	0.000	0	0.009	0.007	1.255	2.10E-01	0.000	0	0.003	0.008	0.391	6.96E-01	0.000	0
**LATERALORBITOFRONTAL_AREA**	-0.006	0.008	-0.756	4.49E-01	0.000	0	0.002	0.007	0.253	8.00E-01	0.000	0	0.008	0.007	1.119	2.63E-01	0.000	0
**LINGUAL_AREA**	0.003	0.010	0.306	7.59E-01	0.000	0	-0.001	0.009	-0.164	8.70E-01	0.000	0	-0.004	0.009	-0.408	6.83E-01	0.000	0
**MEDIALORBITOFRONTAL_AREA**	-0.018	0.008	-2.284	2.24E-02	0.001	0	0.005	0.007	0.700	4.84E-01	0.000	0	0.013	0.007	1.834	6.68E-02	0.000	0
**MIDDLETEMPORAL_AREA**	0.011	0.008	1.393	1.64E-01	0.000	0	0.010	0.007	1.446	1.48E-01	0.000	0	-0.004	0.007	-0.605	5.45E-01	0.000	0
**PARAHIPPOCAMPAL_AREA**	-0.016	0.010	-1.596	1.10E-01	0.000	0	-0.010	0.009	-1.126	2.60E-01	0.000	0	0.036	0.009	3.888	1.02E-04	0.002	0
**PARACENTRAL_AREA**	-0.018	0.010	-1.835	6.65E-02	0.000	0	-0.005	0.009	-0.606	5.45E-01	0.000	0	0.013	0.009	1.412	1.58E-01	0.000	0
**PARSOPERCULARIS_AREA**	0.024	0.010	2.457	1.40E-02	0.001	0	0.012	0.009	1.397	1.62E-01	0.000	0	-0.008	0.009	-0.875	3.81E-01	0.000	0
**PARSORBITALIS_AREA**	0.016	0.009	1.795	7.27E-02	0.000	0	0.002	0.008	0.222	8.24E-01	0.000	0	-0.018	0.008	-2.280	2.26E-02	0.001	0
**PARSTRIANGULARIS_AREA**	0.000	0.010	-0.019	9.85E-01	0.000	0	0.007	0.009	0.781	4.35E-01	0.000	0	-0.002	0.009	-0.264	7.92E-01	0.000	0
**PERICALCARINE_AREA**	0.014	0.011	1.262	2.07E-01	0.000	0	0.009	0.010	0.879	3.79E-01	0.000	0	-0.002	0.010	-0.196	8.45E-01	0.000	0
**POSTCENTRAL_AREA**	-0.016	0.008	-2.082	3.74E-02	0.001	0	-0.021	0.007	-3.081	2.07E-03	0.001	0	0.022	0.007	3.071	2.14E-03	0.001	0
**POSTERIORCINGULATE_AREA**	-0.002	0.009	-0.210	8.33E-01	0.000	0	0.002	0.008	0.239	8.11E-01	0.000	0	0.020	0.008	2.325	2.01E-02	0.001	0
**PRECENTRAL_AREA**	0.004	0.008	0.469	6.39E-01	0.000	0	0.003	0.007	0.430	6.68E-01	0.000	0	-0.004	0.007	-0.573	5.67E-01	0.000	0
**PRECUNEUS_AREA**	0.002	0.008	0.294	7.69E-01	0.000	0	0.000	0.007	-0.013	9.89E-01	0.000	0	0.012	0.007	1.613	1.07E-01	0.000	0
**ROSTRALANTERIORCINGULATE_AREA**	-0.004	0.009	-0.453	6.51E-01	0.000	0	0.002	0.008	0.224	8.23E-01	0.000	0	0.019	0.008	2.389	1.69E-02	0.001	0
**ROSTRALMIDDLEFRONTAL_AREA**	-0.003	0.007	-0.458	6.47E-01	0.000	0	0.004	0.007	0.597	5.50E-01	0.000	0	-0.007	0.007	-0.975	3.30E-01	0.000	0
**SUPERIORFRONTAL_AREA**	0.001	0.007	0.135	8.93E-01	0.000	0	0.006	0.006	0.962	3.36E-01	0.000	0	0.001	0.006	0.152	8.79E-01	0.000	0
**SUPERIORPARIETAL_AREA**	0.019	0.009	2.203	2.76E-02	0.001	0	0.013	0.007	1.730	8.37E-02	0.000	0	-0.003	0.008	-0.340	7.34E-01	0.000	0
**SUPERIORTEMPORAL_AREA**	-0.010	0.008	-1.394	1.63E-01	0.000	0	-0.013	0.007	-1.977	4.81E-02	0.000	0	0.002	0.007	0.267	7.90E-01	0.000	0
**SUPRAMARGINAL_AREA**	0.006	0.008	0.747	4.55E-01	0.000	0	0.004	0.007	0.580	5.62E-01	0.000	0	-0.007	0.007	-0.956	3.39E-01	0.000	0
**FRONTALPOLE_AREA**	0.017	0.010	1.617	1.06E-01	0.000	0	0.007	0.009	0.800	4.23E-01	0.000	0	-0.025	0.009	-2.650	8.07E-03	0.001	0
**TEMPORALPOLE_AREA**	-0.010	0.010	-0.982	3.26E-01	0.000	0	-0.005	0.009	-0.594	5.53E-01	0.000	0	0.006	0.010	0.643	5.21E-01	0.000	0

© 2022 Rakesh D et al. *JAMA Network Open*.

**TRANSVERSETEMPORAL_AREA**	-0.010	0.010	-0.947	3.44E-01	0.000	0	-0.011	0.009	-1.202	2.29E-01	0.000	0	0.001	0.009	0.109	9.13E-01	0.000	0
**INSULA_AREA**	-0.011	0.008	-1.298	1.94E-01	0.000	0	-0.001	0.007	-0.092	9.26E-01	0.000	0	0.016	0.008	2.122	3.39E-02	0.001	0
**THALAMUS_SUB**	0.000	0.007	0.060	9.52E-01	0.000	0	0.006	0.006	0.987	3.24E-01	0.000	0	0.011	0.007	1.615	1.06E-01	0.000	0
**CAUDATE_SUB**	-0.002	0.010	-0.225	8.22E-01	0.000	0	-0.002	0.009	-0.201	8.41E-01	0.000	0	-0.015	0.009	-1.600	1.10E-01	0.000	0
**PUTAMEN_SUB**	-0.019	0.010	-1.950	5.12E-02	0.000	0	-0.023	0.009	-2.671	7.59E-03	0.001	0	0.014	0.009	1.572	1.16E-01	0.000	0
**PALLIDUM_SUB**	-0.012	0.010	-1.204	2.29E-01	0.000	0	-0.001	0.009	-0.156	8.76E-01	0.000	0	0.041	0.009	4.455	8.49E-06	0.002	0
**HIPPOCAMPUS_SUB**	0.014	0.009	1.611	1.07E-01	0.000	0	0.008	0.008	1.061	2.89E-01	0.000	0	-0.005	0.008	-0.561	5.75E-01	0.000	0
**AMYGDALA_SUB**	0.005	0.009	0.555	5.79E-01	0.000	0	0.013	0.008	1.700	8.91E-02	0.000	0	0.019	0.008	2.427	1.53E-02	0.001	0
**ACCUMBENS_SUB**	-0.001	0.009	-0.111	9.12E-01	0.000	0	-0.006	0.008	-0.699	4.84E-01	0.000	0	0.026	0.009	2.979	2.90E-03	0.001	0

Note: Table reports uncorrected p values. Effect size (eta^2) was computed using the t_to_eta2 function from the *effectsize* package in R. Folds represents the number of folds the variable was significant in across discovery and replication samples at pFDR < 0.0125. Model equations included fixed effects of the relevant interaction (including lower order main effects), age, sex, scanner type, and whole-brain volume (for surface area and subcortical volume variables), and the random effect of family.

© 2022 Rakesh D et al. *JAMA Network Open*.

eTable 4. Sensitivity Analysis: Model Output Controlling for Race or Ethnicity

	Cortical thickness variable	B	SE	T	P	Eta²
ADI	Cuneus	-0.038	0.013	-2.914	3.58E-03	0.001
	Lateral occipital	-0.021	0.011	-2.044	4.09E-02	0.001
	Lateral orbitofrontal	-0.043	0.013	-3.312	9.32E-04	0.001
	Lingual	-0.043	0.013	-3.436	5.93E-04	0.002
	Paracentral	-0.046	0.013	-3.682	2.33E-04	0.002
	Pericalcarine	-0.042	0.013	-3.240	1.20E-03	0.001
	Postcentral	-0.027	0.012	-2.289	2.21E-02	0.001
	Precentral	-0.046	0.011	-3.997	6.47E-05	0.002
	Rostral middle frontal	-0.060	0.012	-5.226	1.77E-07	0.004
	Superior parietal	-0.022	0.012	-1.891	5.87E-02	0.000
Income-to-needs	Parahippocampal	0.045	0.014	3.166	1.55E-03	0.001
ADI*Income-to-needs	Cuneus	0.019	0.012	1.596	1.11E-01	0.000
	Lateral occipital	0.023	0.010	2.354	1.86E-02	0.001
	Lateral orbitofrontal	0.041	0.012	3.475	5.14E-04	0.002
	Lingual	0.020	0.011	1.723	8.48E-02	0.000
	Pericalcarine	0.037	0.012	3.131	1.75E-03	0.001
	Insula	0.031	0.012	2.612	9.02E-03	0.001
Educational attainment*Income-to-needs	Cuneus	-0.027	0.011	-2.474	1.34E-02	0.001
	Lateral occipital	-0.016	0.009	-1.867	6.19E-02	0.000
	Lateral orbitofrontal	-0.028	0.011	-2.546	1.09E-02	0.001
	Lingual	-0.028	0.011	-2.642	8.27E-03	0.001
	Parahippocampal	-0.026	0.011	-2.358	1.84E-02	0.001
	Pericalcarine	-0.052	0.011	-4.886	1.05E-06	0.003

Note: Model output has been extracted using the whole sample. Table reports uncorrected p values. Effect size (eta²) was computed using the t_to_eta2 function from the *effectsize* package in R. Folds represents the number of folds the variable was significant in across discovery and replication samples. Model equations

© 2022 Rakesh D et al. *JAMA Network Open*.

included fixed effects ADI, education, income-to-needs, age, sex, scanner type, race/ethnicity, and whole-brain volume (for surface area and subcortical volume variables), and the random effect of family.

© 2022 Rakesh D et al. *JAMA Network Open*.

eTable 5. Model Output for Results for Individual Socioeconomic Status Variables

	ADI						Education						INR					
	B	SE	T	P	Eta2	Folds	B	SE	T	P	Eta2	Folds	B	SE	T	P	Eta2	Folds
BANKS_OF_SUPERIOR_TEMPORAL_SULCUS_THICKNESS	-0.076	0.01	-7.369	1.90E-13	0.0071	10	0.090	0.010	8.740	2.83E-18	0.010	10	0.085	0.010	8.470	2.91E-17	0.009	10
CAUDALANTERIORCINGULATE_THICKNESS	-0.016	0.011	-1.473	1.41E-01	0.0003	0	-0.005	0.011	-0.499	6.18E-01	0.000	0	0.004	0.010	0.392	6.95E-01	0.000	0
CAUDALMIDDLEFRONTAL_THICKNESS	-0.06	0.01	-6.06	1.43E-09	0.0049	10	0.027	0.010	2.747	6.03E-03	0.001	0	0.035	0.010	3.622	2.94E-04	0.002	0
CUNEUS_THICKNESS	-0.146	0.011	-13.35	3.29E-40	0.0226	10	0.126	0.011	11.484	2.80E-30	0.017	10	0.129	0.011	12.140	1.31E-33	0.019	10
ENTORHINAL_THICKNESS	-0.027	0.011	-2.546	1.09E-02	0.0009	0	0.027	0.011	2.490	1.28E-02	0.001	0	0.033	0.010	3.126	1.78E-03	0.001	0
FUSIFORM_THICKNESS	-0.083	0.01	-8.113	5.69E-16	0.0086	10	0.099	0.010	9.701	4.01E-22	0.012	10	0.097	0.010	9.773	1.98E-22	0.012	10
INFERIORPARIETAL_THICKNESS	-0.034	0.009	-3.872	1.09E-04	0.002	5	0.049	0.009	5.642	1.74E-08	0.004	8	0.044	0.008	5.184	2.23E-07	0.004	8
INFERIORTEMPORAL_THICKNESS	-0.052	0.01	-5.179	2.29E-07	0.0035	10	0.085	0.010	8.560	1.35E-17	0.010	10	0.087	0.010	9.031	2.12E-19	0.011	10
ISTHMUSCINGULATE_THICKNESS	-0.012	0.011	-1.06	2.89E-01	0.0001	0	0.015	0.011	1.328	1.84E-01	0.000	0	0.013	0.011	1.183	2.37E-01	0.000	0
LATERALOCCIPITAL_THICKNESS	-0.127	0.009	-13.961	9.10E-44	0.0246	10	0.094	0.009	10.339	6.82E-25	0.014	10	0.114	0.009	12.912	9.38E-38	0.021	10
LATERALORBITOFRONTAL_THICKNESS	-0.103	0.011	-9.65	6.57E-22	0.012	10	0.079	0.011	7.465	9.22E-14	0.007	10	0.090	0.010	8.731	3.07E-18	0.010	10
LINGUAL_THICKNESS	-0.157	0.011	-14.719	2.21E-48	0.0273	10	0.128	0.011	11.991	7.72E-33	0.018	10	0.144	0.010	13.912	1.77E-43	0.024	10
MEDIALORBITOFRONTAL_THICKNESS	-0.057	0.011	-5.324	1.04E-07	0.0037	10	0.019	0.011	1.778	7.54E-02	0.000	0	0.028	0.010	2.758	5.84E-03	0.001	0
MIDDLETEMPORAL_THICKNESS	-0.058	0.009	-6.419	1.46E-10	0.0054	10	0.075	0.009	8.322	1.02E-16	0.009	10	0.072	0.009	8.344	8.47E-17	0.009	10
PARAHIPPOCAMPAL_THICKNESS	-0.108	0.011	-9.851	9.27E-23	0.0125	10	0.116	0.011	10.649	2.70E-26	0.015	10	0.130	0.011	12.321	1.47E-34	0.019	10
PARACENTRAL_THICKNESS	-0.124	0.01	-11.862	3.58E-32	0.0181	10	0.101	0.011	9.621	8.64E-22	0.012	10	0.109	0.010	10.720	1.26E-26	0.015	10
PARSOPERCULARIS_THICKNESS	-0.005	0.01	-0.459	6.46E-01	0	0	0.022	0.010	2.217	2.66E-02	0.001	0	0.019	0.010	1.938	5.27E-02	0.000	0
PARSORBITALIS_THICKNESS	-0.019	0.011	-1.781	7.49E-02	0.0004	0	0.028	0.011	2.670	7.61E-03	0.001	0	0.018	0.010	1.766	7.74E-02	0.000	0
PARSTRIANGULARIS_THICKNESS	-0.036	0.01	-3.632	2.83E-04	0.0017	0	0.023	0.010	2.327	2.00E-02	0.001	0	0.026	0.010	2.733	6.29E-03	0.001	0

© 2022 Rakesh D et al. *JAMA Network Open*.

**PERICALCARINE_THICKNESS**	-0.103	0.011	-9.587	1.19E-21	0.0118	10	0.078	0.011	7.296	3.26E-13	0.007	10	0.081	0.010	7.784	7.96E-15	0.008	10
**POSTCENTRAL_THICKNESS**	-0.109	0.01	-10.96	9.54E-28	0.0154	10	0.095	0.010	9.523	2.21E-21	0.012	10	0.105	0.010	10.924	1.41E-27	0.015	10
**POSTERIORCINGULATE_THICKNESS**	-0.005	0.011	-0.443	6.58E-01	0	0	0.019	0.011	1.795	7.27E-02	0.000	0	0.020	0.010	1.893	5.84E-02	0.000	0
**PRECENTRAL_THICKNESS**	-0.083	0.009	-8.832	1.26E-18	0.0103	10	0.069	0.009	7.332	2.51E-13	0.007	10	0.069	0.009	7.517	6.26E-14	0.007	10
**PRECUNEUS_THICKNESS**	-0.087	0.011	-8.307	1.15E-16	0.009	10	0.079	0.011	7.537	5.36E-14	0.007	10	0.083	0.010	8.143	4.48E-16	0.009	10
**ROSTRALANTERIORCINGULATE_THICKNESS**	0.007	0.011	0.647	5.18E-01	0.0001	0	-0.007	0.011	-0.681	4.96E-01	0.000	0	-0.017	0.010	-1.681	9.28E-02	0.000	0
**ROSTRALMIDDLEFRONTAL_THICKNESS**	-0.083	0.009	-8.81	1.53E-18	0.0102	10	0.049	0.010	5.144	2.75E-07	0.004	9	0.046	0.009	5.047	4.59E-07	0.003	9
**SUPERIORFRONTAL_THICKNESS**	-0.055	0.01	-5.511	3.69E-08	0.004	10	0.034	0.010	3.355	7.98E-04	0.002	0	0.027	0.010	2.765	5.71E-03	0.001	0
**SUPERIORPARIETAL_THICKNESS**	-0.082	0.01	-8.34	8.72E-17	0.009	10	0.067	0.010	6.802	1.11E-11	0.006	10	0.065	0.010	6.824	9.50E-12	0.006	10
**SUPERIORTEMPORAL_THICKNESS**	-0.051	0.01	-5.299	1.20E-07	0.0036	10	0.089	0.010	9.257	2.69E-20	0.011	10	0.081	0.009	8.709	3.69E-18	0.010	10
**SUPRAMARGINAL_THICKNESS**	-0.052	0.008	-6.39	1.76E-10	0.0055	10	0.040	0.008	4.923	8.72E-07	0.003	5	0.039	0.008	4.907	9.42E-07	0.003	10
**FRONTALPOLE_THICKNESS**	-0.013	0.01	-1.193	2.33E-01	0.0002	0	0.039	0.011	3.694	2.23E-04	0.002	0	0.032	0.010	3.181	1.47E-03	0.001	0
**TEMPORALPOLE_THICKNESS**	-0.036	0.01	-3.455	5.53E-04	0.0016	0	0.074	0.010	7.171	8.13E-13	0.007	10	0.060	0.010	6.008	1.96E-09	0.005	10
**TRANSVERSETEMPORAL_THICKNESS**	-0.073	0.011	-6.722	1.92E-11	0.0058	10	0.095	0.011	8.880	8.20E-19	0.010	10	0.097	0.010	9.259	2.63E-20	0.011	10
**INSULA_THICKNESS**	-0.066	0.011	-6.089	1.19E-09	0.0048	10	0.077	0.011	7.148	9.62E-13	0.007	10	0.084	0.010	8.078	7.59E-16	0.008	10
**BANKS_OF_SUPERIOR_TEMPORAL_SULCUS_AREA**	0.038	0.009	4.254	2.12E-05	0.0024	9	0.000	0.009	-0.033	9.74E-01	0.000	0	-0.015	0.009	-1.714	8.65E-02	0.000	0
**CAUDALANTERIORCINGULATE_AREA**	0	0.009	0.019	9.85E-01	0	0	-0.017	0.009	-1.769	7.69E-02	0.000	0	-0.019	0.009	-2.137	3.26E-02	0.001	0
**CAUDALMIDDLEFRONTAL_AREA**	-0.011	0.008	-1.322	1.86E-01	0.0002	0	0.025	0.008	2.984	2.86E-03	0.001	0	0.022	0.008	2.654	7.98E-03	0.001	0
**CUNEUS_AREA**	-0.023	0.009	-2.52	1.17E-02	0.0008	0	-0.026	0.009	-2.812	4.94E-03	0.001	0	-0.010	0.009	-1.114	2.65E-01	0.000	0
**ENTORHINAL_AREA**	-0.058	0.01	-5.719	1.11E-08	0.0043	10	0.041	0.010	4.041	5.37E-05	0.002	7	0.044	0.010	4.436	9.30E-06	0.003	9
**FUSIFORM_AREA**	0.003	0.008	0.352	7.25E-01	0	0	0.019	0.008	2.450	1.43E-02	0.001	0	0.016	0.007	2.114	3.45E-02	0.001	0
**INFERIORPARIETAL_AREA**	0.014	0.008	1.83	6.73E-02	0.0004	0	-0.015	0.008	-1.940	5.24E-02	0.001	0	-0.018	0.007	-2.428	1.52E-02	0.001	0
**INFERIORTEMPORAL_AREA**	-0.032	0.007	-4.297	1.75E-05	0.0024	3	0.028	0.007	3.730	1.93E-04	0.002	6	0.018	0.007	2.558	1.05E-02	0.001	0

© 2022 Rakesh D et al. *JAMA Network Open*.

**ISTHMUSCINGULATE_AREA**	0.017	0.009	1.954	5.07E-02	0.0005	0	-0.045	0.009	-5.200	2.05E-07	0.004	10	-0.044	0.008	-5.301	1.18E-07	0.004	9
**LATERALOCCIPITAL_AREA**	-0.02	0.008	-2.599	9.36E-03	0.0009	0	0.025	0.008	3.162	1.57E-03	0.001	0	0.036	0.008	4.723	2.36E-06	0.003	7
**LATERALORBITOFRONTAL_AREA**	0.006	0.007	0.838	4.02E-01	0.0001	0	-0.020	0.008	-2.725	6.45E-03	0.001	0	-0.011	0.007	-1.521	1.28E-01	0.000	0
**LINGUAL_AREA**	-0.005	0.009	-0.498	6.19E-01	0	0	0.003	0.010	0.286	7.75E-01	0.000	0	0.003	0.009	0.311	7.56E-01	0.000	0
**MEDIALORBITOFRONTAL_AREA**	0.028	0.007	3.821	1.34E-04	0.0019	0	-0.031	0.008	-4.104	4.10E-05	0.002	2	-0.019	0.007	-2.550	1.08E-02	0.001	0
**MIDDLETEMPORAL_AREA**	-0.015	0.007	-2.203	2.76E-02	0.0006	0	0.014	0.007	1.991	4.65E-02	0.001	0	0.011	0.007	1.657	9.76E-02	0.000	0
**PARAHIPPOCAMPAL_AREA**	0.03	0.009	3.193	1.41E-03	0.0013	0	-0.001	0.010	-0.111	9.12E-01	0.000	0	-0.022	0.009	-2.338	1.94E-02	0.001	0
**PARACENTRAL_AREA**	0.03	0.009	3.211	1.33E-03	0.0014	0	-0.025	0.009	-2.634	8.46E-03	0.001	0	-0.014	0.009	-1.564	1.18E-01	0.000	0
**PARSOPERCULARIS_AREA**	-0.023	0.009	-2.569	1.02E-02	0.0009	0	0.000	0.009	0.017	9.86E-01	0.000	0	0.017	0.009	1.868	6.17E-02	0.000	0
**PARSORBITALIS_AREA**	-0.034	0.008	-4.182	2.93E-05	0.0023	6	0.039	0.008	4.804	1.58E-06	0.003	7	0.034	0.008	4.290	1.81E-05	0.002	1
**PARSTRIANGULARIS_AREA**	0.019	0.01	1.956	5.05E-02	0.0005	0	-0.011	0.010	-1.106	2.69E-01	0.000	0	-0.013	0.009	-1.366	1.72E-01	0.000	0
**PERICALCARINE_AREA**	-0.008	0.01	-0.795	4.27E-01	0.0001	0	-0.009	0.010	-0.897	3.70E-01	0.000	0	-0.016	0.010	-1.615	1.06E-01	0.000	0
**POSTCENTRAL_AREA**	-0.005	0.007	-0.654	5.13E-01	0.0001	0	-0.004	0.007	-0.512	6.09E-01	0.000	0	-0.004	0.007	-0.501	6.16E-01	0.000	0
**POSTERIORCINGULATE_AREA**	-0.001	0.009	-0.126	9.00E-01	0	0	-0.036	0.009	-4.147	3.40E-05	0.002	7	-0.028	0.008	-3.296	9.85E-04	0.001	0
**PRECENTRAL_AREA**	-0.028	0.007	-3.805	1.43E-04	0.0019	0	0.014	0.007	1.923	5.45E-02	0.001	0	0.021	0.007	2.965	3.03E-03	0.001	0
**PRECUNEUS_AREA**	-0.022	0.007	-3.007	2.65E-03	0.0012	0	-0.002	0.008	-0.296	7.67E-01	0.000	0	0.007	0.007	0.971	3.32E-01	0.000	0
**ROSTRALANTERIORCINGULATE_AREA**	0.017	0.008	2.143	3.22E-02	0.0006	0	-0.022	0.008	-2.693	7.11E-03	0.001	0	-0.018	0.008	-2.219	2.65E-02	0.001	0
**ROSTRALMIDDLEFRONTAL_AREA**	0.005	0.007	0.763	4.45E-01	0.0001	0	0.019	0.007	2.753	5.92E-03	0.001	0	0.000	0.007	-0.002	9.99E-01	0.000	0
**SUPERIORFRONTAL_AREA**	0.008	0.006	1.26	2.08E-01	0.0002	0	0.007	0.006	1.063	2.88E-01	0.000	0	0.006	0.006	0.921	3.57E-01	0.000	0
**SUPERIORPARIETAL_AREA**	-0.025	0.008	-3.115	1.84E-03	0.0013	0	-0.002	0.008	-0.284	7.76E-01	0.000	0	0.009	0.008	1.182	2.37E-01	0.000	0
**SUPERIORTEMPORAL_AREA**	0.028	0.007	3.997	6.46E-05	0.0021	6	0.008	0.007	1.092	2.75E-01	0.000	0	-0.007	0.007	-0.950	3.42E-01	0.000	0
**SUPRAMARGINAL_AREA**	0	0.007	0.024	9.81E-01	0	0	-0.001	0.008	-0.088	9.30E-01	0.000	0	-0.002	0.007	-0.331	7.41E-01	0.000	0
**FRONTALPOLE_AREA**	-0.037	0.01	-3.908	9.41E-05	0.0021	2	0.044	0.010	4.596	4.38E-06	0.003	9	0.038	0.009	4.014	6.03E-05	0.002	1
**TEMPORALPOLE_AREA**	0.016	0.01	1.66	9.70E-02	0.0004	0	-0.001	0.010	-0.065	9.49E-01	0.000	0	-0.010	0.009	-1.030	3.03E-01	0.000	0
**TRANSVERSETEMPORAL_AREA**	0.028	0.01	2.953	3.16E-03	0.0011	0	-0.012	0.010	-1.195	2.32E-01	0.000	0	-0.008	0.009	-0.871	3.84E-01	0.000	0
**INSULA_AREA**	0.037	0.008	4.817	1.49E-06	0.003	7	-0.037	0.008	-4.765	1.92E-06	0.003	7	-0.046	0.008	-6.098	1.13E-09	0.005	10

© 2022 Rakesh D et al. *JAMA Network Open*.

**THALAMUS_SUB**	0.02	0.007	2.983	2.87E-03	0.0011	0	-0.040	0.007	-5.749	9.34E-09	0.004	10	-0.035	0.007	-5.257	1.51E-07	0.004	10
**CAUDATE_SUB**	-0.016	0.01	-1.629	1.03E-01	0.0003	0	0.028	0.010	2.860	4.25E-03	0.001	0	0.006	0.009	0.635	5.26E-01	0.000	0
**PUTAMEN_SUB**	-0.025	0.009	-2.692	7.12E-03	0.0009	0	0.016	0.009	1.787	7.40E-02	0.000	0	0.012	0.009	1.310	1.90E-01	0.000	0
**PALLIDUM_SUB**	0.013	0.009	1.455	1.46E-01	0.0003	0	-0.007	0.009	-0.791	4.29E-01	0.000	0	-0.005	0.009	-0.528	5.97E-01	0.000	0
**HIPPOCAMPUS_SUB**	-0.023	0.008	-2.735	6.25E-03	0.001	0	0.028	0.008	3.297	9.81E-04	0.001	3	0.025	0.008	3.106	1.91E-03	0.001	0
**AMYGDALA_SUB**	-0.025	0.008	-3.097	1.96E-03	0.0012	0	0.009	0.008	1.066	2.87E-01	0.000	0	0.021	0.008	2.694	7.08E-03	0.001	0
**ACCUMBENS_SUB**	0.016	0.009	1.875	6.08E-02	0.0005	0	-0.027	0.009	-3.063	2.20E-03	0.001	0	-0.027	0.009	-3.094	1.98E-03	0.001	0

Note: Results are from models where individual variables were included (using data from the whole sample) across the same 8862 participants as used in the main analysis. Significant folds were the number of folds in which a variable was significant in both discovery and replication samples at pFDR < 0.0167 (to account for three SES indicators in separate models). Table reports uncorrected p values. Effect size (eta^2) was computed using the t_to_eta2 function from the *effectsize* package in R. Model equations included the specified SES variable of interest, fixed effects of age, sex, scanner type, and whole-brain volume (for surface area and subcortical volume variables), and the random effect of family.

© 2022 Rakesh D et al. *JAMA Network Open*.

eTable 6. Model Output for Results Without Controlling for Whole-Brain Volume for Surface Area Variables (When All 3 Socioeconomic Status Variables Are in the Same Model)

	ADI						Education						INR					
	B	SE	T	P	Eta2	Folds	B	SE	T	P	Eta2	Folds	B	SE	T	P	Eta2	Folds
BANKS_OF_SUPERIOR_TEMPORAL_SULCUS_AREA	-0.007	0.012	-0.573	5.67E-01	0.000	0	0.086	0.013	6.646	3.22E-11	0.006	10	0.056	0.013	4.209	2.59E-05	0.002	0
CAUDALANTERIORCINGULATE_AREA	-0.068	0.013	-5.275	1.36E-07	0.004	5	0.066	0.014	4.739	2.19E-06	0.003	8	0.050	0.014	3.497	4.74E-04	0.002	0
CAUDALMIDDLEFRONTAL_AREA	-0.054	0.012	-4.463	8.20E-06	0.003	4	0.096	0.013	7.380	1.75E-13	0.007	10	0.083	0.013	6.243	4.51E-10	0.005	10
CUNEUS_AREA	-0.089	0.012	-7.247	4.66E-13	0.007	10	0.032	0.013	2.377	1.75E-02	0.001	0	0.053	0.014	3.930	8.55E-05	0.002	1
ENTORHINAL_AREA	-0.079	0.012	-6.385	1.82E-10	0.005	10	0.064	0.013	4.758	1.99E-06	0.003	7	0.058	0.014	4.232	2.34E-05	0.002	3
FUSIFORM_AREA	-0.046	0.012	-3.913	9.18E-05	0.002	0	0.101	0.013	7.902	3.13E-15	0.008	10	0.091	0.013	6.945	4.08E-12	0.006	10
INFERIORPARIETAL_AREA	-0.050	0.011	-4.358	1.33E-05	0.002	2	0.072	0.012	5.824	5.97E-09	0.004	10	0.059	0.013	4.654	3.31E-06	0.003	6
INFERIORTEMPORAL_AREA	-0.087	0.012	-7.518	6.16E-14	0.007	10	0.105	0.013	8.317	1.06E-16	0.009	10	0.072	0.013	5.572	2.60E-08	0.004	9
ISTHMUSCINGULATE_AREA	-0.067	0.012	-5.407	6.59E-08	0.004	7	0.050	0.013	3.752	1.76E-04	0.002	0	0.041	0.014	2.957	3.12E-03	0.001	0
LATERALOCCIPITAL_AREA	-0.056	0.011	-4.994	6.06E-07	0.003	4	0.079	0.012	6.430	1.35E-10	0.005	10	0.098	0.013	7.854	4.57E-15	0.008	10
LATERALORBITOFRONTAL_AREA	-0.066	0.012	-5.484	4.30E-08	0.004	6	0.069	0.013	5.288	1.27E-07	0.004	9	0.083	0.013	6.175	6.96E-10	0.005	10
LINGUAL_AREA	-0.052	0.012	-4.182	2.93E-05	0.002	2	0.068	0.014	4.998	5.93E-07	0.003	9	0.062	0.014	4.467	8.03E-06	0.003	2
MEDIALORBITOFRONTAL_AREA	-0.041	0.012	-3.448	5.67E-04	0.002	0	0.062	0.013	4.762	1.96E-06	0.003	7	0.087	0.013	6.567	5.47E-11	0.006	10
MIDDLETEMPORAL_AREA	-0.073	0.011	-6.427	1.38E-10	0.005	10	0.094	0.012	7.612	3.02E-14	0.007	10	0.079	0.013	6.227	5.00E-10	0.005	10
PARAHIPPOCAMPAL_AREA	-0.019	0.013	-1.550	1.21E-01	0.000	0	0.089	0.014	6.505	8.24E-11	0.005	10	0.040	0.014	2.848	4.42E-03	0.001	0
PARACENTRAL_AREA	-0.024	0.013	-1.891	5.87E-02	0.000	0	0.051	0.014	3.727	1.95E-04	0.002	0	0.075	0.014	5.365	8.33E-08	0.004	7
PARSOPERCULARIS_AREA	-0.071	0.012	-5.764	8.52E-09	0.004	9	0.051	0.013	3.787	1.54E-04	0.002	0	0.080	0.014	5.858	4.87E-09	0.004	10
PARSORBITALIS_AREA	-0.074	0.012	-6.283	3.50E-10	0.005	10	0.103	0.013	8.021	1.20E-15	0.008	10	0.082	0.013	6.273	3.73E-10	0.005	10
PARSTRIANGULARIS_AREA	-0.025	0.012	-2.084	3.72E-02	0.001	0	0.056	0.013	4.205	2.64E-05	0.002	3	0.049	0.014	3.600	3.20E-04	0.002	0
PERICALCARINE_AREA	-0.060	0.013	-4.690	2.78E-06	0.003	5	0.053	0.014	3.802	1.45E-04	0.002	1	0.026	0.014	1.845	6.50E-02	0.000	0
POSTCENTRAL_AREA	-0.068	0.011	-6.023	1.79E-09	0.005	10	0.079	0.012	6.378	1.90E-10	0.005	10	0.069	0.013	5.501	3.90E-08	0.004	8

© 2022 Rakesh D et al. *JAMA Network Open*.

**POSTERIORCINGULATE_AREA**	-0.079	0.012	-6.451	1.18E-10	0.005	10	0.046	0.013	3.450	5.64E-04	0.002	0	0.053	0.014	3.858	1.15E-04	0.002	0
**PRECENTRAL_AREA**	-0.079	0.011	-7.027	2.29E-12	0.006	10	0.078	0.012	6.358	2.16E-10	0.005	10	0.083	0.012	6.666	2.81E-11	0.006	10
**PRECUNEUS_AREA**	-0.090	0.012	-7.487	7.81E-14	0.007	10	0.076	0.013	5.853	5.01E-09	0.004	10	0.084	0.013	6.321	2.75E-10	0.005	9
**ROSTRALANTERIORCINGULATE_AREA**	-0.052	0.012	-4.254	2.12E-05	0.002	1	0.070	0.013	5.234	1.70E-07	0.004	9	0.075	0.014	5.490	4.15E-08	0.004	10
**ROSTRALMIDDLEFRONTAL_AREA**	-0.050	0.011	-4.412	1.04E-05	0.003	3	0.115	0.012	9.416	6.05E-21	0.011	10	0.065	0.012	5.196	2.09E-07	0.003	10
**SUPERIORFRONTAL_AREA**	-0.054	0.012	-4.683	2.87E-06	0.003	4	0.103	0.013	8.197	2.87E-16	0.009	10	0.096	0.013	7.473	8.67E-14	0.007	10
**SUPERIORPARIETAL_AREA**	-0.084	0.012	-7.204	6.40E-13	0.007	10	0.062	0.013	4.906	9.50E-07	0.003	10	0.077	0.013	5.897	3.86E-09	0.004	8
**SUPERIORTEMPORAL_AREA**	-0.029	0.012	-2.548	1.08E-02	0.001	0	0.112	0.013	8.853	1.04E-18	0.010	10	0.077	0.013	6.013	1.91E-09	0.005	10
**SUPRAMARGINAL_AREA**	-0.059	0.011	-5.208	1.96E-07	0.003	7	0.080	0.012	6.525	7.21E-11	0.005	10	0.070	0.013	5.523	3.44E-08	0.004	8
**FRONTALPOLE_AREA**	-0.063	0.012	-5.097	3.53E-07	0.003	8	0.090	0.013	6.730	1.82E-11	0.006	10	0.065	0.014	4.727	2.32E-06	0.003	5
**TEMPORALPOLE_AREA**	-0.027	0.012	-2.155	3.12E-02	0.001	0	0.070	0.014	5.161	2.52E-07	0.003	10	0.047	0.014	3.367	7.64E-04	0.001	0
**TRANSVERSETEMPORAL_AREA**	-0.018	0.013	-1.467	1.42E-01	0.000	0	0.063	0.014	4.584	4.62E-06	0.003	6	0.070	0.014	5.020	5.28E-07	0.003	9
**INSULA_AREA**	-0.041	0.012	-3.502	4.65E-04	0.002	0	0.071	0.013	5.507	3.77E-08	0.004	9	0.045	0.013	3.411	6.50E-04	0.001	0

Note: Model output has been extracted using the whole sample. Significant folds were the number of folds in which a variable was significant in both discovery and replication samples at *p*FDR < 0.0125. Table reports uncorrected p values. Effect size (eta^2) was computed using the t_to_eta2 function from the *effectsize* package in R. Model equations included fixed effects of the three SES variables, age, sex, scanner type, and whole-brain volume (for subcortical volume variables), and the random effect of family.

© 2022 Rakesh D et al. *JAMA Network Open*.

eTable 7. Model Output for Whole-Brain Measures (When All 3 Socioeconomic Status Variables Are in the Same Model)

Independent Variable	Dependent Variable	B	SE	T	P
**Total surface area**					
	ADI	-0.080	0.011	-7.119	1.18E-12
	income_to_needs	0.097	0.012	7.827	5.66E-15
	education_average_years	0.108	0.012	8.848	< 2e-16
**Average cortical thickness**					
	ADI	-0.067	0.012	-5.728	1.05E-08
	income_to_needs	0.041	0.013	3.149	1.64E-03
	education_average_years	0.047	0.013	3.685	2.30E-04
**Whole brain volume**					
	ADI	-0.086	0.011	-7.574	4.04E-14
	income_to_needs	0.109	0.013	8.633	<2e-16
	education_average_years	0.120	0.012	9.668	<2e-16

© 2022 Rakesh D et al. *JAMA Network Open*.

eTable 8. Sensitivity Analysis: Model Output Controlling for Mean Thickness

	Cortical thickness variable	B	SE	T	P	Eta^2
**ADI**	Cuneus	-0.058	0.010	-5.553	2.89E-08	0.004
	Lateral occipital	-0.044	0.007	-6.202	5.88E-10	0.005
	Lateral orbitofrontal	-0.023	0.009	-2.582	9.83E-03	0.001
	Lingual	-0.062	0.010	-6.381	1.86E-10	0.005
	Paracentral	-0.035	0.008	-4.204	2.65E-05	0.002
	Pericalcarine	-0.044	0.011	-4.031	5.61E-05	0.002
	Postcentral	-0.024	0.009	-2.839	4.54E-03	0.001
	Precentral	-0.011	0.007	-1.618	1.06E-01	0.000
	Rostral middle frontal	-0.026	0.007	-3.925	8.76E-05	0.002
	Superior parietal	-0.006	0.007	-0.978	3.28E-01	0.000
**Income-to-needs**	Parahippocampal	0.065	0.013	4.871	1.13E-06	0.003
**ADI*Income-to-needs**	Cuneus	0.041	0.010	4.185	2.89E-05	0.002
	Lateral occipital	0.045	0.007	6.796	1.16E-11	0.006
	Lateral orbitofrontal	0.033	0.008	3.927	8.66E-05	0.002
	Lingual	0.041	0.009	4.413	1.03E-05	0.002
	Pericalcarine	0.049	0.010	4.757	2.00E-06	0.003
	Insula	0.032	0.010	3.187	1.44E-03	0.001
**Educational attainment*Income-to-needs**	Cuneus	-0.036	0.009	-4.031	5.61E-05	0.002
	Lateral occipital	-0.024	0.006	-3.950	7.87E-05	0.002
	Lateral orbitofrontal	-0.014	0.008	-1.837	6.63E-02	0.000
	Lingual	-0.034	0.009	-4.027	5.71E-05	0.002
	Parahippocampal	-0.034	0.010	-3.281	1.04E-03	0.001
	Pericalcarine	-0.059	0.010	-6.163	7.49E-10	0.005

© 2022 Rakesh D et al. *JAMA Network Open*.

eTable 9. Sensitivity Analysis: Model Output Including Site as a Random Effect

	Cortical thickness variable	B	SE	T	P	Eta²
ADI	Cuneus	-0.098	0.015	-6.508	8.2E-11	0.006
	Lateral occipital	-0.096	0.013	-7.680	1.8E-14	0.008
	Lateral orbitofrontal	-0.066	0.015	-4.516	6.4E-06	0.003
	Lingual	-0.110	0.015	-7.483	8.3E-14	0.009
	Paracentral	-0.075	0.014	-5.213	2E-07	0.007
	Pericalcarine	-0.069	0.015	-4.675	3E-06	0.003
	Postcentral	-0.088	0.014	-6.384	2E-10	0.014
	Precentral	-0.043	0.013	-3.345	0.00083	0.004
	Rostral middle frontal	-0.032	0.013	-2.427	0.01527	0.001
	Superior parietal	-0.066	0.014	-4.830	1.4E-06	0.004
Income-to-needs	Parahippocampal	0.073	0.014	5.155	2.6E-07	0.003
ADI*Income-to-needs	Cuneus	0.038	0.012	3.151	0.00163	0.001
	Lateral occipital	0.046	0.010	4.646	3.4E-06	0.003
	Lateral orbitofrontal	0.043	0.012	3.673	0.00024	0.002
	Lingual	0.045	0.012	3.848	0.00012	0.002
	Pericalcarine	0.041	0.012	3.517	0.00044	0.002
	Insula	0.035	0.012	2.979	0.0029	0.001
Educational attainment*Income-to-needs	Cuneus	-0.031	0.011	-2.875	0.00406	0.001
	Latera loccipital	-0.029	0.009	-3.226	0.00126	0.001
	Lateral orbitofrontal	-0.032	0.011	-2.996	0.00275	0.001
	Lingual	-0.039	0.011	-3.636	0.00028	0.002
	Parahippocampal	-0.041	0.011	-3.720	0.0002	0.002
	Pericalcarine	-0.050	0.011	-4.703	2.6E-06	0.003

© 2022 Rakesh D et al. *JAMA Network Open*.

eTable 10. Sensitivity Analysis: Weighted Model Output (Accounting for Rescaled Propensity Scores)

	Cortical thickness variable	B	SE	T	P	Eta^2
ADI	Cuneus	-0.093	0.013	-7.313	2.88E-13	0.007
	Lateral occipital	-0.085	0.011	-8.015	1.27E-15	0.008
	Lateral orbitofrontal	-0.071	0.012	-5.720	1.11E-08	0.004
	Lingual	-0.098	0.012	-7.922	2.67E-15	0.008
	Paracentral	-0.083	0.012	-6.856	7.66E-12	0.006
	Pericalcarine	-0.076	0.013	-6.026	1.76E-09	0.005
	Postcentral	-0.063	0.012	-5.412	6.41E-08	0.004
	Precentral	-0.062	0.011	-5.587	2.39E-08	0.004
	Rostral middle frontal	-0.075	0.011	-6.792	1.19E-11	0.006
	Superior parietal	-0.058	0.011	-5.100	3.47E-07	0.003
Income-to-needs	Parahippocampal	0.083	0.015	5.682	1.37E-08	0.004
ADI*Income-to-needs	Cuneus	0.069	0.013	5.439	5.51E-08	0.003
	Lateral occipital	0.077	0.010	7.456	9.83E-14	0.007
	Lateral orbitofrontal	0.067	0.012	5.432	5.72E-08	0.003
	Lingual	0.070	0.012	5.692	1.30E-08	0.004
	Pericalcarine	0.073	0.012	5.923	3.29E-09	0.004
	Insula	0.057	0.013	4.572	4.90E-06	0.002
Educational attainment*Income-to-needs	Cuneus	-0.066	0.011	-5.807	6.59E-09	0.004
	Latera loccipital	-0.059	0.009	-6.241	4.55E-10	0.005
	Lateral orbitofrontal	-0.050	0.011	-4.497	6.98E-06	0.002
	Lingual	-0.065	0.011	-5.819	6.14E-09	0.004
	Parahippocampal	-0.052	0.012	-4.495	7.06E-06	0.002
	Pericalcarine	-0.086	0.011	-7.625	2.69E-14	0.007

© 2022 Rakesh D et al. *JAMA Network Open*.

eReferences

1. Morrissey TW, Vinopal KM. Neighborhood Poverty and Children's Academic Skills and Behavior in Early Elementary School. *J Marriage Fam*. 2018;80(1):182-197. doi:10.1111/jomf.12430
2. Gordon RA, Savage C, Lahey BB, et al. Family and neighborhood income: Additive and multiplicative associations with youths' well-being. *Soc Sci Res*. 2003;32(2):191-219. doi:10.1016/S0049-089X(02)00047-9
3. Saragosa-Harris NM, Chaku N, MacSweeney N, et al. A practical guide for researchers and reviewers using the ABCD Study and other large longitudinal datasets. *Dev Cogn Neurosci*. 2022;55:101115. doi:10.1016/J.DCN.2022.101115
4. Panizzon MS, Fennema-Notestine C, Eyler LT, et al. Distinct Genetic Influences on Cortical Surface Area and Cortical Thickness. *Cereb Cortex*. 2009;19(11):2728-2735. doi:10.1093/cercor/bhp026
5. Tamnes CK, Bos MGN, van de Kamp FC, Peters S, Crone EA. Longitudinal development of hippocampal subregions from childhood to adulthood. *Dev Cogn Neurosci*. 2018;30(November 2017):212-222. doi:10.1016/j.dcn.2018.03.009
6. Gogtay N, Giedd JN, Lusk L, et al. Dynamic mapping of human cortical development during childhood through early adulthood. *Proc Natl Acad Sci U S A*. 2004;101(21):8174-8179. doi:10.1073/pnas.0402680101
7. Wierenga LM, Langen M, Oranje B, Durston S. Unique developmental trajectories of cortical thickness and surface area. *Neuroimage*. 2014;87:120-126. doi:10.1016/j.neuroimage.2013.11.010
8. Heeringa SG, Berglund PA. A Guide for Population-based Analysis of the Adolescent Brain Cognitive Development (ABCD) Study Baseline Data. *bioRxiv*. Published online February 10, 2020:2020.02.10.942011. doi:10.1101/2020.02.10.942011
9. Taylor RL, Cooper SR, Jackson JJ, Barch DM. Assessment of Neighborhood Poverty, Cognitive Function, and Prefrontal and Hippocampal Volumes in Children. *JAMA Netw open*. 2020;3(11):e2023774. doi:10.1001/jamanetworkopen.2020.23774
10. Marek S, Tervo-Clemmens B, Nielsen AN, et al. Identifying reproducible individual differences in childhood functional brain networks: An ABCD study. *Dev Cogn Neurosci*. 2019;40:100706. doi:10.1016/j.dcn.2019.100706
11. Hackman DA, Cserbik D, Chen J-CC, et al. Association of Local Variation in Neighborhood Disadvantage in Metropolitan Areas with Youth Neurocognition and Brain Structure. *JAMA Pediatr*. Published online May 3, 2021:e210426. doi:10.1001/jamapediatrics.2021.0426

© 2022 Rakesh D et al. *JAMA Network Open*.
